# Host immune network-guided reverse vaccinology approach for prioritizing candidate antigens in *Schistosoma japonicum*–associated liver fibrosis

**DOI:** 10.1371/journal.pntd.0013519

**Published:** 2026-08-17

**Authors:** Qianxu Yang, Jue Wang, Yingzi Ming, Yu Zhang, Li Ping Wong, Hai Yen Lee

**Affiliations:** 1 Centre for Population Health (CePH), Department of Social and Preventive Medicine, Faculty of Medicine, Universiti Malaya, Kuala Lumpur, Malaysia; 2 Faculty of Chinese Medicine and State Key Laboratory of Quality Research in Chinese Medicine, Macau University of Science and Technology, MacauChina; 3 Center for Organ Transplantation, The Third Xiangya Hospital, Central South University, Changsha, Hunan, China; 4 Key Laboratory of Translational Research in Transplantation Medicine of National Health Commission, Third Xiangya Hospital, Central South University, Changsha, Hunan, China; 5 Hunan Province Clinical Research Center for Infectious Diseases, Changsha, Hunan, China; 6 Department of Epidemiology and Health Statistics, School of Public Health, Fujian Medical University, Fuzhou, Fujian, China; 7 Department of Medicine, College of Medicine, Korea University, Seongbuk-gu, Seoul, Republic of Korea; 8 Tropical Infectious Diseases Research and Education Centre, Universiti Malaya, Kuala Lumpur, Malaysia; Cairo University, EGYPT

## Abstract

*Schistosoma japonicum*–associated liver fibrosis (SSLF) remains a major cause of long-term morbidity in schistosomiasis, even in post-control or low-transmission settings. While most vaccine development efforts have focused on reducing parasite burden, few approaches have incorporated host pathology related to liver fibrosis. This study developed an in silico strategy that combined host single-cell analysis with reverse vaccinology to guide the exploratory screening of parasite antigens with potential relevance to SSLF. Single-cell transcriptomics of fibrotic and control human liver tissues was used to identify immune-related molecular networks and host proteins associated with fibrosis-related pathology in the available dataset. In parallel, antigens were screened by functional annotation and established reverse-vaccinology criteria. Candidate antigens were further assessed by docking against selected host proteins. Docking of a known FYN inhibitor was included as a structural reference to interpret the strongest predicted antigen–host interaction. Single-cell analyses identified FYN, BCL2, and AKT3 as key nodes in fibrosis-associated immune networks. Among screened parasite antigens, DRE2_SCHJA, an Anamorsin homolog involved in iron–sulfur cluster assembly, emerged as a computational candidate for further validation. Docking predicted that DRE2_SCHJA may bind in an SH2-associated region of FYN, whereas the Saracatinib–FYN model was centered on the ATP-binding pocket of the kinase domain. This difference may suggest a distinct predicted binding mode rather than Saracatinib-like kinase inhibition. Overall, the vaccine-screening profile and predicted FYN-associated interaction may provide a computational rationale for further experimental evaluation of DRE2_SCHJA as a candidate antigen in the immune–fibrotic context of SSLF.

## Author summary

Schistosomiasis remains a major public health problem because it can cause long-term organ damage even after parasite control. Liver fibrosis is one of its most serious complications and is driven by chronic immune responses to parasite eggs. However, most schistosomiasis vaccine research has focused on reducing infection or parasite burden, with less attention to the tissue damage that causes long-term disease. In this study, we combined reverse vaccinology with immune features from fibrotic liver single-cell data to guide parasite-antigen prioritization. DRE2_SCHJA was prioritized as an exploratory candidate antigen with possible relevance to schistosomiasis-associated liver fibrosis. Because a schistosomiasis-only liver single-cell dataset was unavailable, the findings should be viewed as hypothesis-generating rather than as proof of protection or antifibrotic efficacy. Experimental studies are still needed to evaluate DRE2_SCHJA as an antigen candidate. By linking parasite-antigen screening with liver pathology, this approach may help guide more disease-relevant candidate selection for future vaccine research.

## Introduction

Schistosomiasis control in China has entered a low-transmission and post-control phase, with sustained mass drug administration and surveillance substantially reducing active infections caused by *Schistosoma japonicum* (*S. japonicum*) [1]. However, chronic morbidity persists among previously exposed individuals. *Schistosoma japonicum*–associated liver fibrosis (SSLF) results from prolonged egg deposition in the hepatic portal system, which drives granulomatous inflammation and progressive fibrogenesis [2]. Praziquantel clears adult worms and remains central to control programs, but it does not reliably reverse established fibrosis or fully address chronic hepatic pathology once disease has developed [3]. Consequently, some previously exposed individuals continue to face risks of chronic liver damage, portal-hypertension-related complications, and long-term disability [4]. In post-control settings, SSLF therefore remains an important source of morbidity that cannot be fully addressed by chemotherapy alone.

Despite continued efforts, progress in schistosomiasis vaccine development has been limited [5]. To date, only four candidates (Sh28GST, Sm14, Sm-TSP-2, and Sm-p80) have advanced to clinical trials, and these have been evaluated primarily for their ability to reduce infection intensity or parasite burden [6]. Most studies have focused on *Schistosoma mansoni* (*S. mansoni*) or *Schistosoma haematobium* (*S. haematobium*), whereas vaccine development for *S. japonicum* remains comparatively underexplored. Clinical and preclinical assessments have also emphasized immunogenicity and parasitological endpoints rather than long-term pathological outcomes. For example, Sh28GST elicited measurable immune responses and reductions in infection-related indicators, but did not provide sustained protection against disease recurrence in field trials [7]. These findings suggest that immunogenicity and infection-related endpoints remain important, but may be insufficient to capture long-term disease outcomes in endemic settings. Recent studies have therefore emphasized that future schistosomiasis vaccines should move beyond infection prevention and consider chronic disease manifestations [8]. However, how to incorporate pathology-relevant targets into vaccine prioritization remains insufficiently defined, particularly for SSLF.

SSLF is driven by sustained host responses to parasite eggs retained in the hepatic portal system [9]. Egg antigens induce a dominant Th2-skewed immune environment, characterized by prolonged cytokine production and chronic inflammation [10]. Over time, this response activates hepatic stellate cells, increases extracellular matrix deposition, and drives progressive fibrogenesis [11]. SSLF is therefore not caused by a single cell type or pathway. Instead, it reflects interactions among immune cells, such as T lymphocytes and macrophages, and resident stromal cells in the liver. These immune–stromal interactions connect inflammation with fibrotic remodeling and shape disease progression [12].

Host signaling molecules involved in immune-cell activation and survival have been linked to inflammation, hepatic stellate-cell activation, and extracellular matrix remodeling during chronic liver injury [13]. Evidence from non-parasitic fibrosis models also shows that Src-family kinases and related signaling nodes can be experimentally modulated [14]. These findings support the use of host signaling nodes to interpret parasite-antigen candidates in relation to immune-driven fibrosis. Because SSLF reflects coordinated immune and stromal responses to *S. japonicum* infection, parasite-antigen prioritization may need to consider host immune networks involved in liver fibrogenesis, rather than conventional vaccine-related properties alone.

This study used host molecular networks related to liver fibrogenesis to support computational antigen prioritization. Single-cell transcriptomic data from fibrotic and control liver tissues were examined to identify immune-related host networks with potential relevance to SSLF. We then screened *S. japonicum* antigens using established reverse vaccinology criteria. Candidate antigens were docked with selected host hub proteins to predict possible antigen–host interactions. For the top-scoring antigen–host pair, we compared the predicted binding pattern with the docking pattern of a known inhibitor of the same host protein, using the inhibitor model as a structural reference. This strategy may help prioritize parasite proteins with potential relevance to SSLF for future experimental evaluation.

## Materials and methods

### Identification of SSLF-associated hub-gene candidates

**Fig 1:**
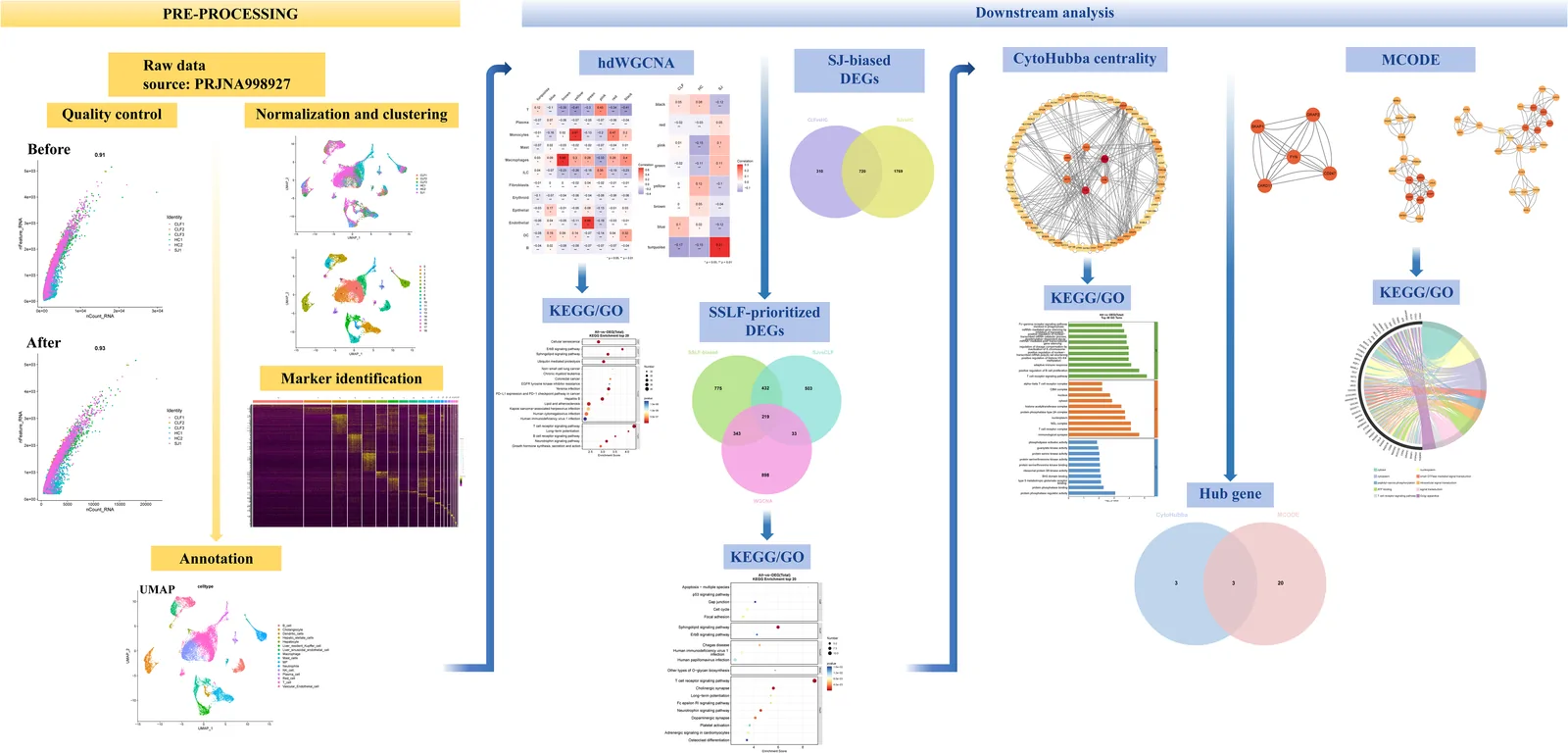
Integrated single-cell and network-analysis workflow for prioritizing SSLF-associated hub-gene candidates.

The workflow for identifying SSLF-associated hub genes is outlined in Fig 1, integrating single-cell transcriptomics, co-expression network, differential expression, and PPI analyses.

### Single-cell RNA sequencing analysis

Single-cell RNA sequencing (scRNA-seq) data were obtained from the PRJNA998927 dataset, originally generated by Yu Zhang *et al.* (2021), comprising liver tissues from *S. japonicum*–HBV co-infection (SJ, n = 1), HBV-induced fibrosis (CLF, n = 3), and healthy controls (HC, n = 2). Raw reads were processed with CeleScope (v2.0.7) for barcode filtering and UMI deduplication and aligned using STAR (v2.7.3a) [15]. To contextualize the use of this publicly available dataset, the following epidemiological considerations are relevant.

In the current epidemiological setting, schistosomiasis in China has entered a low-prevalence and near-elimination phase, making human liver tissues from patients with SSLF difficult to obtain. Co-infection with HBV or other locally prevalent infections is also not uncommon among individuals with schistosomiasis [16]. Even when patients with schistosomiasis-associated liver disease are identified, liver tissue suitable for scRNA-seq is rarely available because liver transplantation cases involving schistosomiasis-associated liver disease are limited [17], and routine diagnostic liver biopsy specimens often provide insufficient tissue quantity and cell viability for single-cell transcriptomic analysis. Accordingly, publicly available human liver scRNA-seq datasets for schistosomiasis-associated fibrosis are scarce, and, to our knowledge, the dataset analyzed here was the only relevant resource available at the time of analysis. We therefore used this dataset with a comparative, multi-step filtering and prioritization workflow designed to enrich for SSLF-associated signals and prioritize a focused set of candidate hub genes.

Single-cell data processing and alignment were performed as follows. Cells were filtered in Seurat (v4.3.0) based on gene count (200–10,000) and mitochondrial content (< 25%) [19], with doublets removed by DoubletFinder (v2.0.3). Normalization and identification of highly variable genes were followed by PCA, clustering, and dimensionality reduction (UMAP/t-SNE). Cell types were annotated using SingleR (v2.0.0) and refined by manual inspection of cluster-specific marker genes, with highly similar clusters merged for downstream analysis.

### High-dimensional weighted gene co-expression network analysis (HD-WGCNA)

To construct gene co-expression modules from scRNA-seq data using metacell aggregation, gene co-expression modules were identified using HD-WGCNA (v0.3.01) on a Harmony-integrated Seurat object [18]. Genes expressed in ≥ 5% of cells were retained, and metacells were constructed per cell type and condition using k-nearest neighbors (k = 25, max_shared = 10) on Harmony-reduced dimensions. A signed hybrid network was built with a soft-thresholding power of 3 (scale-free R² ≈ 0.95, mean connectivity ≈ 30). Modules were detected by hierarchical clustering of the TOM and refined with dynamic tree cutting. To identify WGCNA modules preferentially associated with the SJ sample (*S. japonicum* condition) compared with CLF (HBV-induced fibrosis) and HC (healthy control) samples, module eigengenes (MEs) and memberships (kME) were calculated, and module-trait associations with condition (HC, CLF, SJ) were assessed using Pearson correlation (p < 0.05). To identify the cell population most strongly associated with the *S. japonicum*–associated WGCNA module, module activity across cell populations was quantified using UCell, and module–cell type associations were evaluated by Pearson correlation (p < 0.05). Modules showing significant associations with the *S. japonicum* condition were subsequently subjected to Gene Ontology and KEGG pathway enrichment analysis using the clusterProfiler package.

### Differentially expressed genes (DEGs)

Following the identification of the *S. japonicum*–associated WGCNA module, the cell population showing the strongest module–trait association with SJ sample was prioritized for downstream analyses. Differential expression analysis was subsequently performed within this cell population using Seurat’s FindMarkers (adjusted p < 0.05, |log_2_FC| ≥ 1) [19] to compare SJ vs. HC, CLF vs. HC, and SJ vs. CLF. To account for transcriptional changes associated with HBV-related fibrosis in the co-infected SJ sample, CLF was included as a disease-background control.

To reduce the influence of HBV-related fibrosis, DEGs identified from the SJ vs. HC comparison were first filtered to exclude genes also differentially expressed in the CLF vs. HC comparison. The resulting SJ-biased genes were then intersected with DEGs from the SJ vs. CLF comparison and with genes from the SJ-associated WGCNA module, yielding a prioritized gene set relevant to SSLF. To assess whether the SSLF-prioritized gene set exhibited functional themes distinct from those observed in HBV-only fibrosis, GO and KEGG enrichment analyses were performed in parallel for CLF–HC DEGs (adjusted p < 0.05, |log_2_FC| ≥ 1), SJ–HC DEGs (adjusted p < 0.05, |log_2_FC| ≥ 1), and the SSLF-prioritized gene set to compare their functional profiles.

### Protein-protein interaction network (PPI) construction and module analysis

Protein–protein interaction (PPI) networks for the SSLF-prioritized candidate DEGs were constructed based on STRING interactions, utilizing a confidence score ≥0.4 [20]. Hub gene prioritization was performed using the cytoHubba plugin in Cytoscape (v3.10.3) by ranking nodes across 12 centrality metrics, including Maximal Clique Centrality (MCC), Density of Maximum Neighborhood Component (DMNC), Maximum Neighborhood Component (MNC), Degree, Edge Percolated Component (EPC), BottleNeck, EcCentricity, Closeness, Radiality, Betweenness, Stress, and Clustering Coefficient [21]. Genes showing consistently high centrality across these metrics were considered central PPI nodes. Separately, densely connected subnetworks were identified using the MCODE plugin based on the same set of interaction-connected PPI nodes, with default parameters (degree cutoff = 2, node score cutoff = 0.2, k-core = 2, maximum depth = 100; haircut enabled, fluff disabled). The top three modules were selected based on MCODE scores. Because MCODE identifies multiple densely connected subnetworks, genes from the top three MCODE modules were combined into a single gene set. SSLF-associated hub genes were then defined as genes shared between this combined MCODE set and the top-ranked central nodes identified by CytoHubba [22]. The complete analytical workflow leading to the identification of the SSLF-associated genes is summarized in S1 Table. Enrichment analyses were performed separately for genes in the connected PPI network and for genes in the top three MCODE modules to capture functional features at the global network and modular levels.

### Identification of potential antigens against SSLF

**Fig 2:**
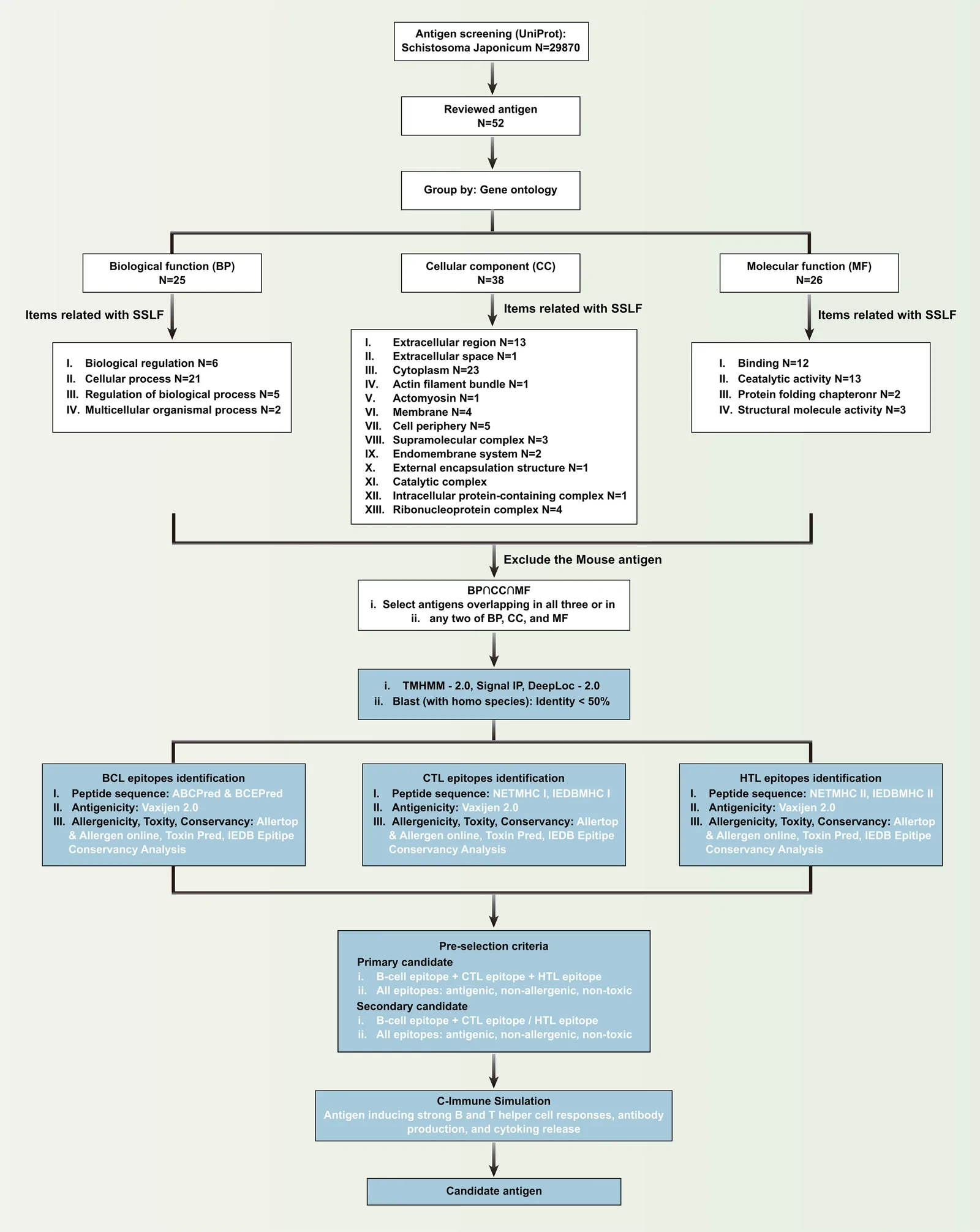
Reverse vaccinology workflow for prioritizing SSLF-associated candidate antigens.

The antigen screening workflow (Fig 2) included filtering reviewed *S. japonicum* proteins by GO annotations, structural features, and host similarity, followed by B-cell, CTL, and HTL epitope prediction and safety assessment. Peptides meeting both immunogenicity and safety criteria were retained as SSLF vaccine candidates.

### Antigen selection based on GO annotation and homology exclusion

Reviewed *S. japonicum* proteins were retrieved from UniProt and filtered by Gene Ontology (GO) terms related to liver fibrosis. Antigens annotated in at least two GO domains (Biological Process, Cellular Component, Molecular Function) were retained. Transmembrane helices, signal peptides, and subcellular localization were predicted using TMHMM 2.0 (https://services.healthtech.dtu.dk/services/TMHMM-2.0/), SignalP 5.0 (https://services.healthtech.dtu.dk/services/SignalP-5.0/), and DeepLoc 2.0 (https://services.healthtech.dtu.dk/services/DeepLoc-2.0/), respectively. These predictions were used as contextual information during antigen prioritization and were not applied as exclusion criteria [23]. This approach reflects the fact that immune accessibility is not confined to surface-exposed or secreted proteins. It also avoided overlooking potentially relevant antigens due to reliance on predicted subcellular localization as a primary filtering criterion. Initial antigen screening prioritized sequence similarity to human proteins, assessed by BLAST, and retained only candidates showing <50% identity to human sequences [24].

### BCL epitope prediction

Linear B-cell epitopes were predicted using ABCPred (https://webs.iiitd.edu.in/raghava/abcpred/index.html) and BCEPRED (https://webs.iiitd.edu.in/raghava/bcepred/index.html) under default settings. In ABCPred, a window length of 16 amino acids and the default score threshold of 0.51 were applied for initial prediction, and high-confidence candidates with scores ≥ 0.85 were retained for downstream analysis [25]. BCEPRED was applied using hydrophilicity (threshold = 2.0), flexibility (1.9), accessibility (2.0), and antigenic propensity (1.8), all under default threshold settings. Candidate B-cell epitope regions were then identified through a consensus-based approach, retaining sequence segments supported by at least two of these properties. These regions were further filtered by requiring overlap with high-confidence ABCPred 16-mer predictions (score ≥ 0.85). Candidate regions identified by both Bcepred and ABCPred were further evaluated for antigenicity using VaxiJen v2.0 (https://www.ddg-pharmfac.net/vaxijen/VaxiJen/VaxiJen.html), with the target organism set as parasite and a threshold of 0.5 [26].

### CTL epitope prediction

Cytotoxic T lymphocyte (CTL) epitopes were predicted using NetMHCpan-4.1 (https://services.healthtech.dtu.dk/services/NetMHCpan-4.1/) and the IEDB MHC-I binding tool (https://tools.iedb.org/mhci/). For NetMHCpan-4.1, predictions were performed against HLA-A and HLA-B class I alleles prevalent in the Chinese population, including HLA-A*02:01, HLA-A*02:07, HLA-A*11:01, HLA-A*24:02, HLA-B*46:01, HLA-B*40:01, HLA-B*58:01, and HLA-B*15:01 [27]. In NetMHCpan-4.1, peptides classified as strong binders (SB; %rank ≤ 0.5) were retained, while weak binders (WB; %rank ≤ 2.0) were included only when no strong binders were identified for a given allele. All other parameters were kept at default settings, and no additional %rank filtering was applied (filtering threshold set to −99). The IEDB MHC-I binding tool was applied using the previously described HLA-A and HLA-B alleles, as well as additional HLA-C alleles commonly reported in the Chinese population, including HLA-C*06:02, HLA-C*01:02, and HLA-C*03:02. Peptides with a binding rank ≤1% in IEDB were retained [28]. Only peptides predicted by both NetMHCpan-4.1 and IEDB MHC-I were assessed for antigenicity via VaxiJen v2.0, with the target organism set as parasite and a threshold of 0.5 [26].

### HTL epitope prediction

Helper T lymphocyte (HTL) epitopes were identified using NetMHCII (https://services.healthtech.dtu.dk/services/NetMHCII-2.3/) and IEDB MHC-II tools (https://tools.iedb.org/mhcii/). For NetMHCII analysis, HLA class II alleles prevalent in the Chinese population were selected. These included HLA-DRB1*03:01, DRB1*07:01, DRB1*09:01, DRB1*12:01, DRB1*15:01, and representative DQA1–DQB1 pairs (HLA-DQA1*02:01–DQB1*02:02, DQA1*02:01–DQB1*03:01, DQA1*02:01–DQB1*03:03, DQA1*02:01–DQB1*04:02, DQA1*03:01–DQB1*03:01, DQA1*03:01–DQB1*03:02, and DQA1*03:03–DQB1*04:02). Predictions were performed using a peptide length of 15 amino acids, with all other parameters kept at the default settings of the NetMHCII server. In parallel, HTL epitopes were predicted using the IEDB MHC-II binding tool with the NetMHCIIpan 4.1 EL algorithm (version 2023.09). The same peptide length (15 amino acids) and HLA class II alleles as those used in NetMHCII analysis were applied, and all other parameters were kept at default settings. Selection followed the same criteria as applied in CTL epitope prediction. Only peptides predicted by both NetMHCII and the IEDB MHC-II tools were retained and subsequently evaluated for antigenicity using VaxiJen v2.0 [26].

### Allergy, toxicity and conservancy prediction

Allergenicity was assessed with AllerTOP 2.0 (https://www.ddg-pharmfac.net/allertop_test/) and AllergenOnline (http://www.allergenonline.org/databasefasta.shtml). Toxicity was evaluated with ToxinPred3.0 (https://webs.iiitd.edu.in/raghava/toxinpred3/prediction.php), and epitope conservancy was analyzed with the IEDB Epitope Conservancy Tool (https://tools.iedb.org/conservancy/) [29]. All analyses were performed using the default parameters provided by each tool.

### Antigen selection criteria

Antigens were defined as primary candidates if they contained at least one B-cell epitope and both CTL and HTL epitopes, with all meeting the criteria of antigenicity, non-allergenicity (as predicted by both allergenicity tools), non-toxicity, and 100% sequence conservancy. Antigens meeting these criteria but containing only one type of T-cell epitope (either CTL or HTL) were considered secondary candidates [30]. This classification strategy prioritized antigens with favorable immunogenic and safety profiles for subsequent validation.

### Immune simulation of antigens

In silico immune simulations of candidate antigens were performed using C-ImmSim (https://150.146.2.1/C-IMMSIM/index.php?page=0) [31]. C-ImmSim is a rule-based, mechanistic simulation framework designed to approximate relative immune response patterns under defined assumptions, rather than to provide quantitative or predictive estimates of protective efficacy. Immune simulations were conducted with default C-ImmSim settings. The simulation volume was set to 10, with 100 simulation steps. A single vaccine injection (without LPS) was administered at time step 1, using an antigen dose of 1000 units and an adjuvant level of 100. A fixed random seed (12345) was applied. A representative set of HLA class I and class II alleles commonly reported in Chinese populations relevant to schistosomiasis-endemic regions was selected for C-ImmSim simulations. Accordingly, the simulation included HLA-A*02:07, HLA-A*11:01, HLA-B*46:01, HLA-B*58:01, HLA-DRB1*03:01, and HLA-DRB1*09:01 [32]. Simulation outputs were interpreted based on multiple immune components. These outputs were used to assess the internal consistency and relative trends of simulated immune responses.

### Docking and computational analyses

#### First protein–protein docking.

We used protein–protein docking to assess interactions between *S. japonicum* antigens and host fibrosis-related hub proteins. Structures were obtained from UniProt, prioritizing high-resolution data or AlphaFold models. Non-protein molecules were removed in PyMOL (v2.5.3). Docking was conducted using ClusPro (default parameters) [33], with resulting complexes ranked by ClusPro scores and binding affinities predicted by PRODIGY [34] (https://rascar.science.uu.nl/prodigy/) with default parameters at 25 °C. Docked complexes were first evaluated based on PRODIGY-predicted binding free energy (ΔG), with ClusPro docking scores used to support ranking when ΔG values were comparable. The top three ranked complexes were selected for further structural inspection in PyMOL. Subsequently, interfacial features, including hydrogen bonds, salt bridges, and π–π stacking interactions, were examined qualitatively to characterize interface features and to support candidate prioritization. Docking analyses were conducted using a standardized workflow and interpreted in a comparative, hypothesis-generating context, with results considered in light of the limitations of static structural models.

### Molecular docking

Molecular docking was performed in Maestro (v13.9, Schrödinger LLC) to generate a reference inhibitor–hub protein complex for structural comparison with subsequent antigen–protein docking analyses [35]. The protein was prepared with the Protein Preparation Wizard (hydrogen addition, bond order assignment, H-bond optimization, and restrained minimization using the OPLS4 force field). Based on the protein docking analyses described above, FYN was selected as a representative hub protein and docked with the clinically investigated FYN inhibitor Saracatinib as a reference interaction. Saracatinib was prepared using LigPrep to generate low-energy conformations and appropriate protonation states at physiological pH (7.0 ± 0.5). Docking was conducted with Glide in extra precision (XP) mode. Docking poses were ranked by docking score, and the top-ranked pose was inspected to assess whether the predicted inhibitor binding site was consistent with reported Saracatinib–FYN interaction sites. This reference docking provided a theoretically informed binding interface, which was subsequently used for qualitative comparison with antigen–hub protein docking results.

### Secondary protein–protein docking

Because the FYN structure used in the first protein–protein docking differed from the Saracatinib-bound FYN structure, the same antigen was re-docked to this inhibitor-bound FYN structure. All docking settings, ClusPro parameters, and ranking criteria were kept identical to those applied in the first protein–protein docking. This secondary docking was performed to enable qualitative comparison of the antigen–protein binding interface with the reference site defined by the inhibitor–hub protein complex under a consistent conformational framework.

### Interface visualization

Docking results were visualized by aligning the hub protein structures in PyMOL (v2.5.3) and mapping the binding sites of the inhibitor and the antigen to compare their spatial distribution.

### Motif prediction

To explore antigen–host protein interactions, short linear motifs (SLiMs) in the antigen sequence were predicted using the Eukaryotic Linear Motif (ELM) resource (http://elm.eu.org). No subcellular compartment filtering was applied, as the antigen’s subcellular context relevant to host protein engagement is not established in silico. Analysis was performed with the taxonomic context set to *Schistosoma japonicum* and other parameters kept at default settings. Predicted motifs were used to highlight putative functional elements with potential relevance to protein binding and regulatory interactions [36].

## Results

### Single-cell RNA sequencing analysis

**Fig 3:**
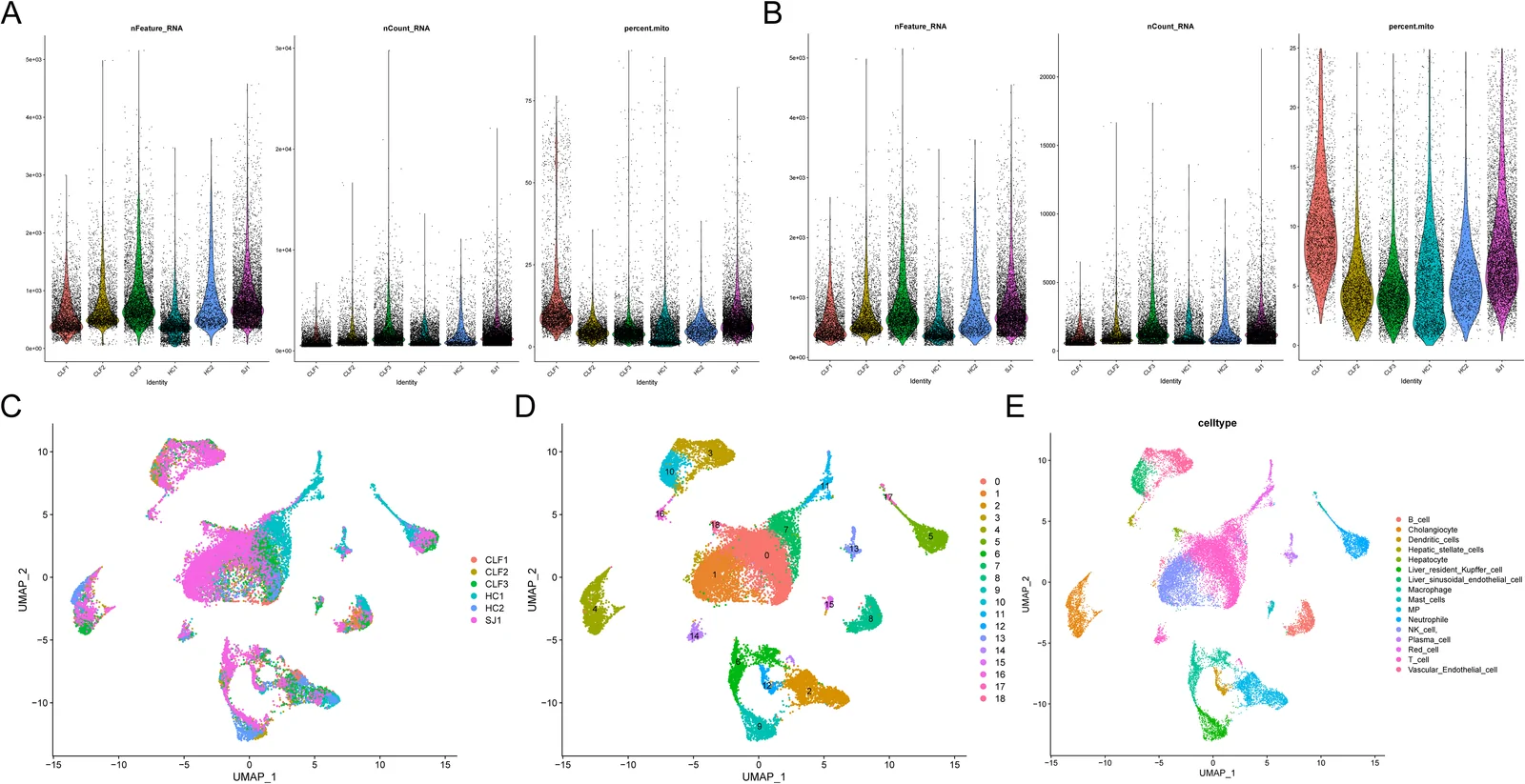
Quality control, clustering, and cell-type annotation of human liver single-cell transcriptomes across SJ, CLF, and HC samples. (A) Violin plots of quality-control metrics before filtering, including the number of detected genes per cell, total UMI counts per cell, and the percentage of mitochondrial transcripts. (B) Violin plots of the same quality-control metrics after filtering. (C) UMAP of cells by sample origin. (D) UMAP of 19 clusters. (E) UMAP annotation of 16 major cell types. SJ, *S. japonicum*–HBV co-infection; CLF, HBV-induced fibrosis; HC, healthy controls.

**Fig 4:**
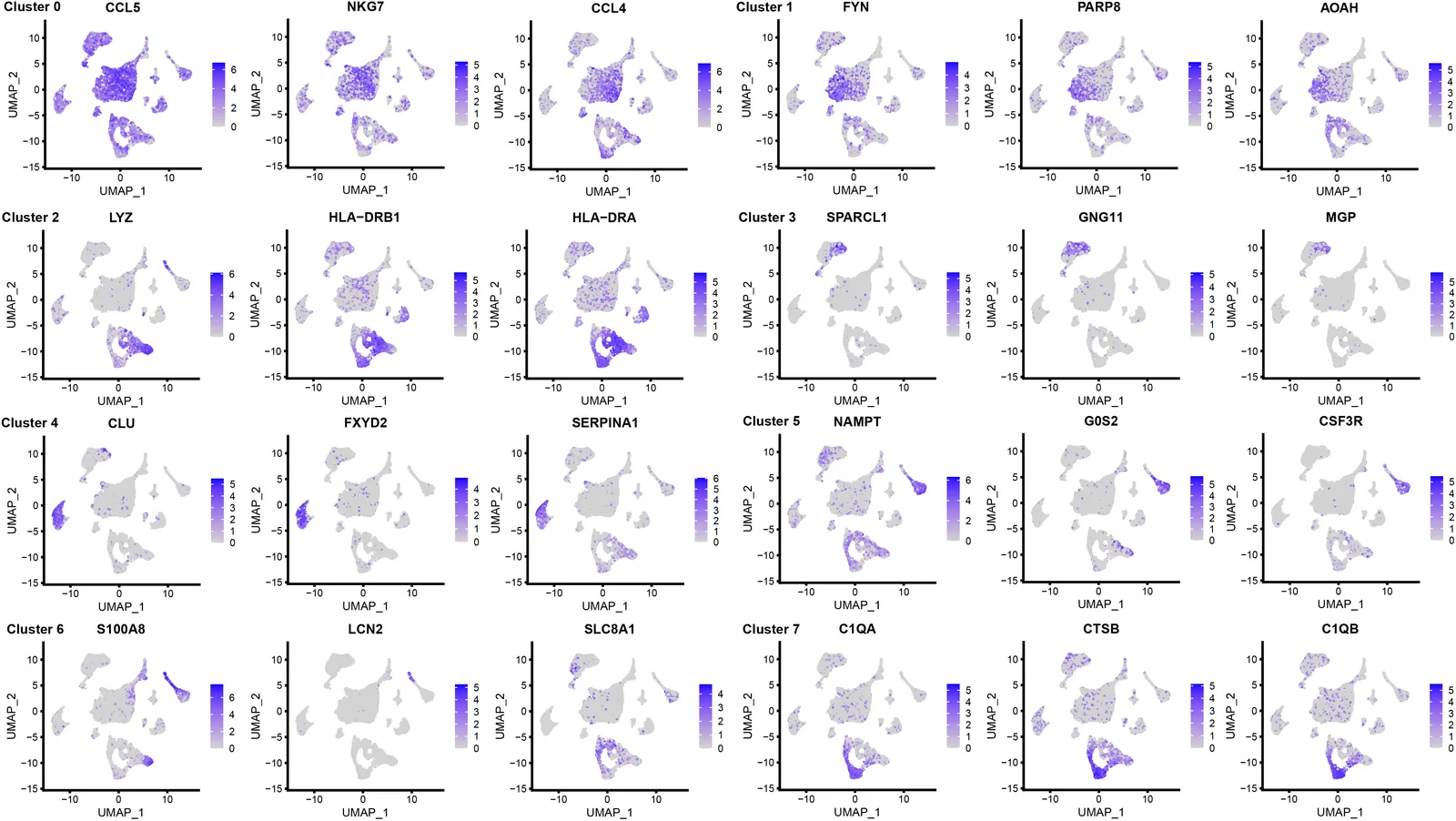
UMAP feature plots of the top three marker genes for clusters 0–7. Color intensity represents normalized expression level.

Single-cell RNA sequencing was performed on six liver samples, generating high-quality data with valid read rates exceeding 96% and Q30 scores above 93% (S1 Fig). Barcode rank plots clearly distinguished high-quality cells from background noise based on UMI count distribution (S2 Fig). The median number of genes detected per cell ranged from 389 to 752, and sequencing saturation was < 92% across samples. Quality control metrics confirmed high data quality, with most cells expressing hundreds to thousands of genes and < 20% mitochondrial content (Fig 3A and 3B). After this, 22,672 cells were retained. Unsupervised clustering identified 19 clusters (Fig 3C and 3D). Guided by marker expression and reference-based annotation, these clusters were annotated and consolidated into 16 major cell populations (Fig 3E), including T cells, NK cells, macrophages, Kupffer cells, hepatic stellate cells, hepatocytes, and other immune and parenchymal populations. Fig 4 displays the top marker genes of clusters 0–7, while the remaining annotated populations are provided in S3 Fig.

### High-dimensional weighted gene co-expression network analysis (HD-WGCNA) and enrichment analysis

**Fig 5:**
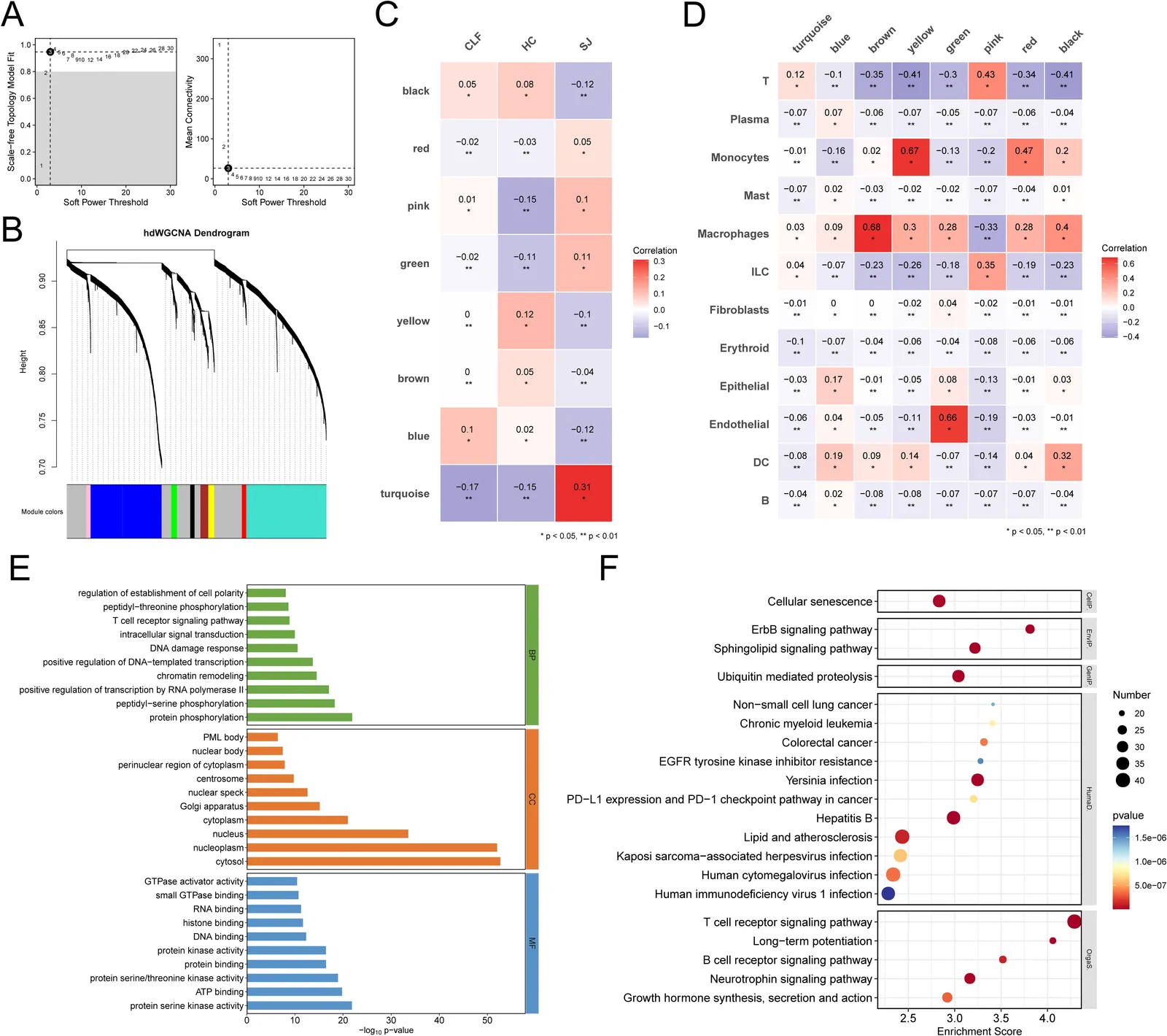
hdWGCNA-based analysis and functional annotation of gene modules associated with SSLF. (A) Soft-thresholding power selection for hdWGCNA network construction. (B) Gene dendrogram showing module assignment by color. (C) Module–trait correlation heatmap across HBV-induced fibrosis (CLF), healthy control (HC) and *S. japonicum*–HBV co-infection liver fibrosis (SJ) samples. (D) Module–cell type correlation heatmap between gene modules and annotated cell types. (E) GO enrichment of genes in the module most correlated with SJ sample. (F) KEGG enrichment of genes in the module most correlated with SJ sample.

Using scale-free topology, β = 3 was chosen for network construction (Fig 5A). HD-WGCNA identified eight modules from 3,432 genes (Fig 5B). Module sizes varied across the network, with the turquoise module being the largest (n = 1,493), followed by the blue module (n = 1,329), while the remaining modules contained between 80 and 147 genes (black, n = 82; brown, n = 147; green, n = 108; pink, n = 80; red, n = 82; yellow, n = 111). Among all identified modules, the turquoise module showed the strongest association with SJ samples (r = 0.31, p < 0.05; Fig 5C). Among all immune cell types, T cells displayed the highest correlation with the turquoise module (r = 0.12, p < 0.05; Fig 5D). In this dataset, the turquoise module was most associated with the SJ sample and was characterized by T cell–related transcriptional features. Therefore, the turquoise module was prioritized for downstream functional enrichment analyses. GO and KEGG enrichment analyses highlighted functional categories related to protein phosphorylation, cytosolic localization, and kinase activity, as well as immune-associated pathways, with the T cell receptor signaling pathway being the most prominently enriched (Fig 5 E and 5F).

### DEGs

**Fig 6:**
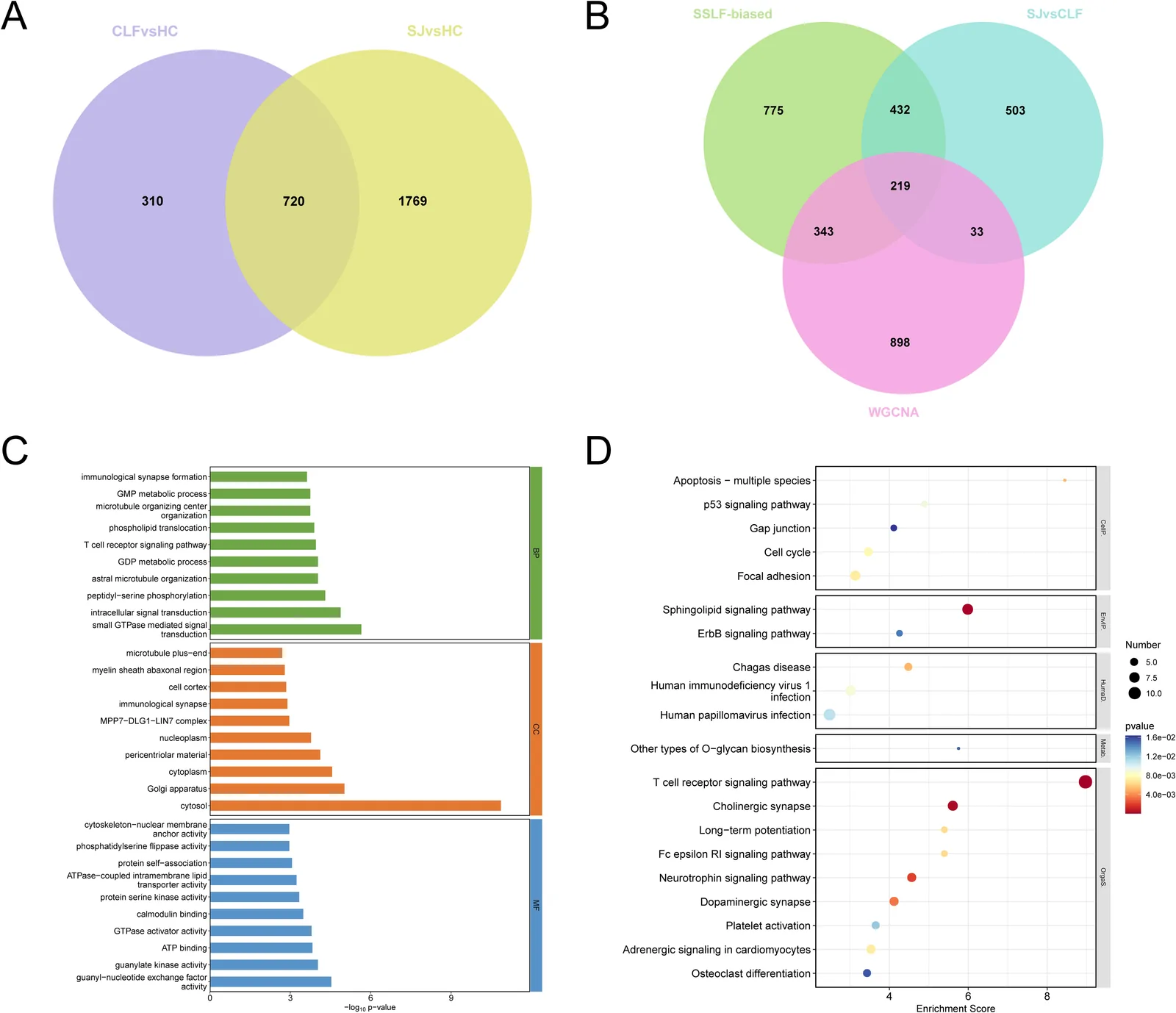
DEG prioritization and functional enrichment of SSLF-associated candidate genes. (A) Venn diagram of DEGs from CLF vs. HC and SJ vs. HC comparisons. (B) Venn diagram showing the overlap among SSLF-biased DEGs, SJ vs. CLF DEGs, and genes from the hdWGCNA turquoise module. SSLF-biased DEGs refer to the SJ vs. HC DEGs that did not overlap with CLF vs. HC DEGs. (C) GO enrichment of the overlapping genes identified in panel B. (D) KEGG enrichment of the overlapping genes identified in panel B.

Based on HD-WGCNA, the turquoise module showed the strongest association with *S. japonicum* sample and the highest correlation with T cells. Therefore, subsequent differential expression analysis focused on T cells. Across the three pairwise comparisons, 2,489 DEGs were identified in SJ–HC (*S. japonicum*–HBV co-infection), 1,030 from CLF–HC (HBV-only), and 1,187 in SJ–CLF. After excluding genes shared between the SJ–HC and CLF–HC DEG sets, 1,769 SJ-biased DEGs were retained (Fig 6A). Intersection of this SJ-biased DEG set (n = 1,769) with DEGs from the SJ–CLF comparison (n = 1,187) and genes assigned to the *S. japonicum*–associated turquoise WGCNA module (n = 1,493) yielded 219 SSLF-prioritized candidate genes (Fig 6B).

To assess whether the SSLF-prioritized gene set exhibited functional patterns distinct from HBV-only fibrosis, GO and KEGG enrichment analyses were performed in parallel for T-cell DEGs from CLF–HC (HBV-only), SJ–HC (*S. japonicum*–HBV co-infection), and the final SSLF-prioritized gene set (Figs 6C and 6D, S4). In the CLF–HC contrast, enrichment was dominated by an extracellular remodeling program. GO terms highlighted angiogenesis, cell adhesion, and collagen-containing extracellular matrix (S4A Fig). KEGG pathways identified complement and coagulation cascades, ECM–receptor interaction, and PI3K–Akt signaling as the main enriched pathways (S4C Fig) [37]. Overall, this profile is consistent with chronic HBV-associated fibrotic remodeling. In the co-infection contrast (SJ–HC), enrichment showed a heterogeneous immune–inflammatory profile. GO terms included immune response, T cell receptor signaling and general signal transduction (S4B Fig), while KEGG analysis highlighted NF-κB and T cell receptor signaling pathways (S4D Fig). This pattern is consistent with combined parasitic and viral immune activation rather than a single dominant pathological program. By contrast, the SSLF-prioritized gene set showed a functional shift toward T cell–linked regulation and intracellular signaling (Fig 6C and 6D). GO enrichment emphasized intracellular, small GTPase–mediated signaling and immune synapse–related processes, while KEGG analysis highlighted T cell receptor and sphingolipid signaling pathways. Importantly, pathways dominant in the HBV-only contrast, including extracellular matrix remodeling and complement/coagulation cascades, were no longer among the leading enriched signatures. The SSLF-prioritized gene set showed an enrichment pattern centered on immune-regulatory signals rather than extracellular matrix remodeling. This pattern is more consistent with SSLF-related immunopathology [13].

### PPI, MCODE and hub gene identification

**Fig 7:**
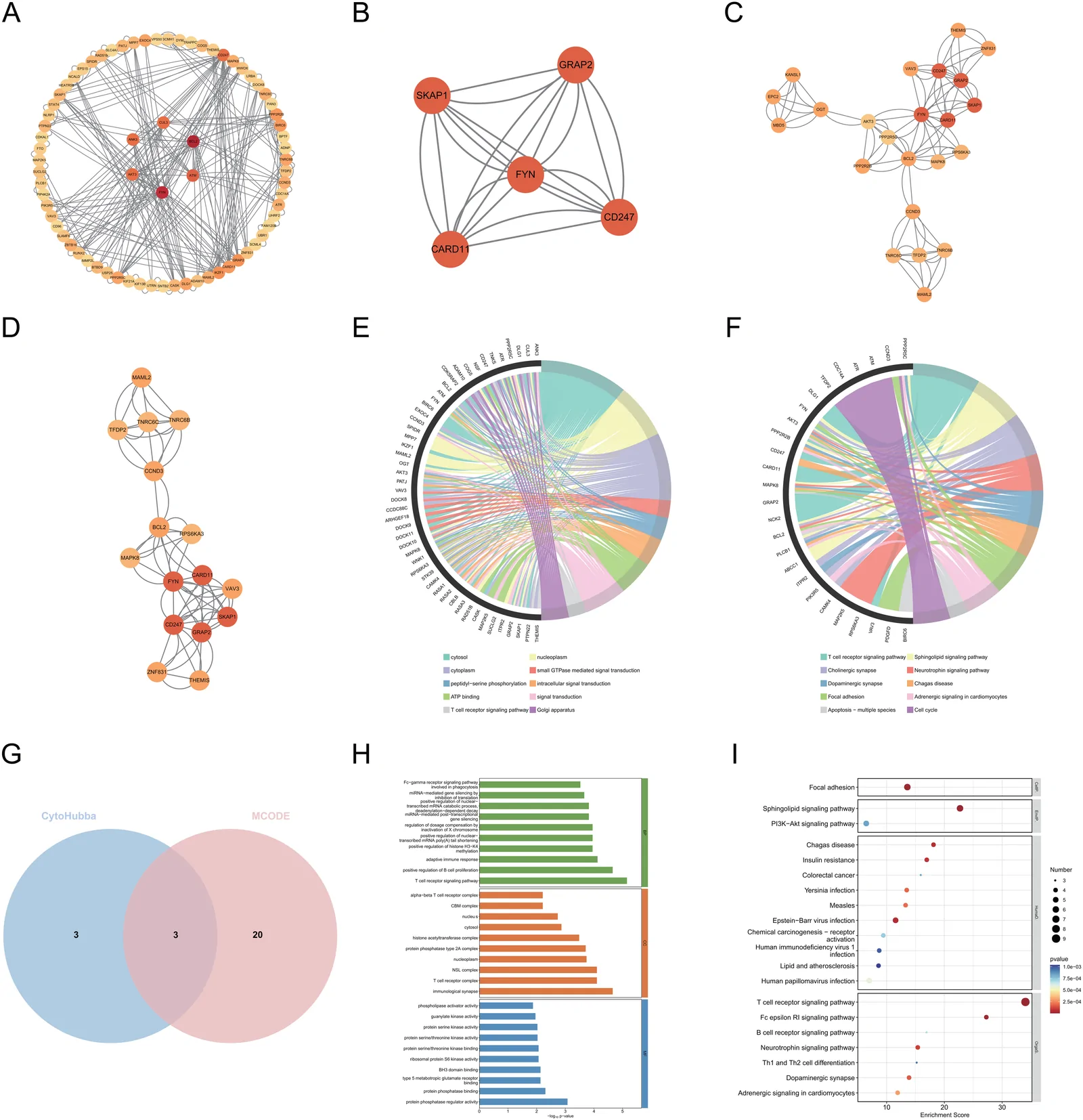
PPI network-based prioritization of SSLF-associated hub-gene candidates. (A) CytoHubba centrality analysis of the STRING-derived PPI network. (B-D) Top three MCODE modules. (E, F) GO and KEGG enrichment of all genes included in the CytoHubba centrality network. (G) Overlap of CytoHubba-prioritized central genes with genes combined from the top three MCODE modules. (H, I) GO and KEGG enrichment analysis of genes combined from the top three MCODE modules.

Among the 219 SSLF-prioritized candidate genes, 152 showed documented protein–protein interaction relationships and were used to construct a STRING-based PPI network (S5 Fig). Network centrality analysis using the cytoHubba plugin ranked nodes across twelve topological metrics, from which six genes were consistently prioritized as the most central nodes in the network: AKT3, ANK3, ATM, BCL2, CUL3, and FYN (Fig 7A). In parallel, the same 152-node PPI network was subjected to MCODE clustering analysis, which identified three densely connected subnetworks. The top-scoring module (Cluster 1) exhibited an MCODE score of 5.0 and comprised 5 genes connected by 20 edges (Fig 7B). Cluster 2 had an MCODE score of 4.545 and contained 23 genes with 100 edges (Fig 7C), while Cluster 3 showed an MCODE score of 4.667 and included 16 genes connected by 70 edges (Fig 7D). Because MCODE modules represent distinct dense subnetworks rather than parallel ranking criteria, genes from the top three modules were merged into a single module-derived gene set (23 unique genes). Intersection of this set with the cytoHubba-prioritized central genes (n = 6) identified three SSLF hub candidates: AKT3, FYN, and BCL2 (Fig 7G). GO terms of the connected PPI network (n = 152) were enriched in cytosol and nucleoplasm, while KEGG pathways highlighted T cell receptor and sphingolipid signaling (Fig 7E and 7F). Module-specific enrichment (n = 23) further emphasized immune-related functions, including T cell receptor signaling and immunological synapse pathways (Fig 7H and 7I). Both network-level and module-level enrichment analyses pointed to T cell–related signaling pathways.

### Antigen screening

From UniProt, 29,870 *S. japonicum* entries were retrieved, including 52 reviewed entries (S2 Table). GO annotation showed that 25 antigens had annotations in at least two of the three GO domains: biological process (BP), cellular component (CC), and molecular function (MF) (S3 Table). Of these, 14 had annotations in all three domains, whereas 11 had annotations in two domains (S6 Fig). As shown in Table 1, all 25 candidates had known subcellular localization categories in this analysis. Among them, 22 were assigned to intracellular compartments, including the cytoplasm, nucleus, and/or mitochondrion. Six of these 22 candidates showed dual intracellular localization assignments, involving either cytoplasm–mitochondrion or cytoplasm–nucleus localization. The remaining three of the 25 candidates showed secretory-pathway or extracellular features: PPIB_SCHJA was predicted to contain both a transmembrane helix and a signal peptide, consistent with endoplasmic reticulum localization; CYSP_SCHJA carried a signal peptide and was predicted to be extracellular; and HGLB_SCHJA was predicted to be extracellular despite the absence of a signal peptide. BLAST analysis against the human proteome identified sixteen low-homology candidates (<50% identity): DRE2_SCHJA, GST28_SCHJA, ANX13_SCHJA, SORCN_SCHJA, MYSP_SCHJA, CIAO1_SCHJA, HGLB_SCHJA, KYNU_SCHJA, ATAT_SCHJA, EIF3M_SCHJA, GST26_SCHJA, CYSP_SCHJA, CDO_SCHJA, FABP_SCHJA, TCTP_SCHJA, and RSSA_SCHJA. These candidates were therefore retained for subsequent analyses.

**Table 1:** BLAST-based screening and predicted features of *Schistosoma japonicum* antigens.

Antigen	TMHMM 2.0	SignalP 5.0	DeepLoc 2.0	BLAST (%)
PPIE_SCHJA	0	Nucleus	Nucleus	62.5%
PPIB_SCHJA	1	0.970780	Endoplasmic reticulum, Signal peptide	59.7%
HGLB_SCHJA	0	0.2436	Extracellular	39.6%
DRE2_SCHJA	0	0	Nucleus	29.1%
PURA_SCHJA	0	0	Cytoplasm	53.8%
ENO_SCHJA	0	0	Cytoplasm	71.2%
KYNU_SCHJA	0	0	Cytoplasm	38.6%
TPIS_SCHJA	0	0	Cytoplasm, Mitochondrion	63.1%
RL24_SCHJA	0	0	Cytoplasm, Mitochondrion	56.8%
RS3A_SCHJA	0	0	Cytoplasm	59%
RSSA_SCHJA	0	0	Cytoplasm	46.8%
CH10_SCHJA	0	0	Mitochondrion	64%
ATAT_SCHJA	0	0	Nucleus	32.1%
EIF3M_SCHJA	0	0	Cytoplasm, Nucleus	32.5%
GST28_SCHJA	0	0	Cytoplasm	28.8%
GST26_SCHJA	0	0	Cytoplasm	38.5%
CYSP_SCHJA	0	0.9986	Extracellular, Signal peptide	37.7%
CDO_SCHJA	0	0	Cytoplasm	37.6%
HSP70_SCHJA	0	0	Cytoplasm	66.5%
ANX13_SCHJA	0	0	Cytoplasm, Nucleus	22.3%
FABP_SCHJA	0	0	Cytoplasm	45%
TCTP_SCHJA	0	0	Cytoplasm, Nucleus	45.9%
SORCN_SCHJA	0	0	Cytoplasm	27.1%
MYSP_SCHJA	0	0	Cytoplasm	18.4%
CIAO1_SCHJA	0	0	Cytoplasm, Nucleus	20.9%

### B-cell epitope identification

Among the 16 antigens retained in the previous screening step using the < 50% sequence identity threshold, six antigens (DRE2_SCHJA, HGLB_SCHJA, KYNU_SCHJA, CYSP_SCHJA, FABP_SCHJA, and TCTP_SCHJA) contained at least one conserved B cell epitope predicted to be antigenic (VaxiJen scores ranging from 0.70 to 1.99), non-allergenic, and non-toxic (Table 2).

**Table 2:** Predicted antigenicity, allergenicity, and toxicity of BCL, CTL, and HTL epitopes.

Antigen	BCL				CTL				HTL			
	Epitopes with antigenicity (VaxiJen score)	Allergenicity	Toxicity	Conservancy	Epitopes with antigenicity (VaxiJen score)	Allergenicity	Toxicity	Conservancy	Epitopes with antigenicity (VaxiJen score)	Allergenicity	Toxicity	Conservancy
DRE2_SCHJA	EATEVED (0.8567)	No	No	100%	No	–	–	–	FIAFVPS (1.8160)	No	No	100%
GST28 _SCHJA	NMMGDTD (1.5311)	Probable allergen or not allergen	No	100%	No	–	–	–	No	–	–	–
ANX13_SCHJA	KGGGTD (1.6695)	Probable allergen or not allergen	No	100%	No	–	–	–	No	–	–	–
SORCN_SCHJA	SRVDADKSGS (1.1509)	Probable allergen or allergen	No	100%	No	–	–	–	No	–	–	–
MYSP_SCHJA	1.EMEQRLRERDEELES (1.3569) 2.TELEETQSRV (0.5016) 3.AEFDGESRRC (0.7942)	Epitopes 1: Non-allergen or allergen Epitopes 2: Probable allergen or allergen 3: Probable allergen or allergen	No	100%	RVDELTIEV (0.9727)	Allergen Or non-allergen	No	100%	No	–	–	–
CIAO1_SCHJA	No	–	–	–	No	–	–	–	No	–	–	
HGLB_ SCHJA	1.IDYKGKKVN (1.0403)	Probable non-allergen	No	100%	No	–	–	–	No	–	–	–
KYNU_SCHJA	1.KFYRPTN (1.1589) 2.LVTPSNSNER (1.0749)	Probable non-allergen	No	100%	No				No	–	–	–
ATAT_SCHJA	QRKGYG (1.7380)	Probable allergen	No	100%	LLSDIPV (0.8700)	Probable allergen	No	100%	No	–	–	–
EIF3M_SCHJA	DNIRRHK (0.7425)	Probable allergen		100%	1.FLAKHPNFL (0.8466) 2.KLFPLNLLF (1.3530)	1. Non-allergen 2. Probable allergen	Yes (FLAKHPNFL)	100%	No	–	–	–
GST26_SCHJA	No	–	–	–	No	–	–	–	No	–	–	–
CYSP_SCHJA	1.ILMGARKEDAE (1.0082) 2.MKRNRR (1.9875)	1. Allergen 2.Non allergen	No	100%	MYGPVEAAF (0.9370)	Probable allergen	–	–	No	–	–	–
CDO_SCHJA	NLIDEG (1.8987)	Probable allergen			No	–	–	–	No	–	–	–
FABP_SCHJA	1.EEFDEKTSD (0.6958) 2.SESKITQTQKDAK (1.3044)	Non-allergen	No	100%	No	–	–	–	No	–	–	–
TCTP_SCHJA	1.IAANPSGEEGQEE (1.0806) 2.VSTSFDKKS (0.8422)	1.Probable allergen 2.Probable non-allergen	No	100%	No	–	–	–	No	–	–	–
RSSA_SCHJA	FLYRDPNEEE (0.7138)	Probable allergen	No	100%	No	–	–	–	No	–	–	–

“No” indicates that the epitope was predicted to be non-antigenic, non-allergenic, or non-toxic, depending on the corresponding assessment. “-” means that the prediction was not performed because the epitope did not meet the preceding selection criteria.

### CTL epitope identification

CTL epitope prediction identified four antigenic CD8 ⁺ T cell epitopes from MYSP_SCHJA (RVDELTIEV), ATAT_SCHJA (LLSDIPV), EIF3M_SCHJA (FLAKHPNFL; KLFPLNLLF), and CYSP_SCHJA (MYGPVEAAF) (Table 2). However, these epitopes were predicted to be allergenic and/or toxic.

### HTL epitope identification

DRE2_SCHJA was the only antigen predicted to contain an HTL epitope (FIAFVPS) fulfilling all criteria of antigenicity (VaxiJen score = 1.82), non-allergenicity, non-toxicity, and full conservancy, whereas no HTL epitopes were identified in the other candidates (Table 2).

### Antigen selection

No candidate antigen simultaneously contained qualifying B-cell, CTL, and HTL epitopes under the predefined immunogenicity and safety criteria. Because no antigen met the full requirement for all three epitope classes, we next considered candidates that retained a qualifying B-cell epitope together with at least one qualifying T-cell epitope class, either CTL or HTL. DRE2_SCHJA was the only antigen that retained a conserved B cell epitope (EATEVED, 222–228) and at least one T cell epitope (HTL; FIAFVPS, 134–140) while satisfying all safety and conservancy criteria. DRE2_SCHJA was therefore retained for subsequent analyses. Functional annotation based on UniProt identified DRE2_SCHJA as an Anamorsin homolog. DRE2_SCHJA is annotated as an intracellular protein involved in the iron–sulfur (Fe–S) cluster assembly pathway, which may support the maturation of Fe–S–dependent proteins involved in parasite metabolism and survival [38,39].

### Immune simulation of antigen

**Fig 8:**
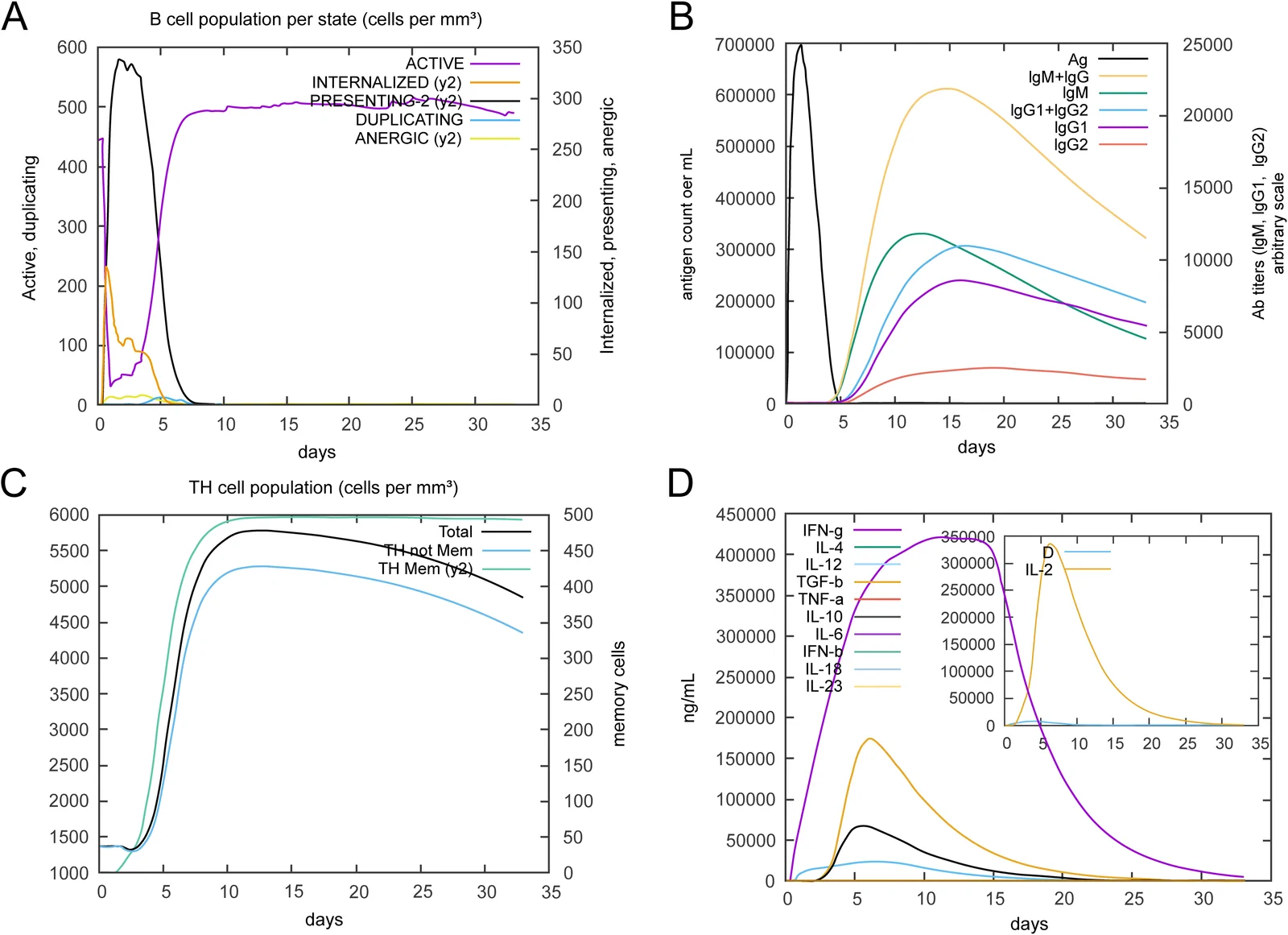
Immune simulation induced by DRE2_SCHJA using the C-ImmSim server. (A) Expansion of active B cells (purple) following antigen stimulation. (B) Antigen clearance and total IgM + IgG production (orange) over time. (C) Induction of memory T helper cells (Green) following vaccination. (D) Cytokine release dominated by IFN-γ (purple) and IL-2 (yellow) peaks after immunization.

As shown in Fig 8, C-ImmSim predicted an immune-response profile for DRE2_SCHJA under the defined simulation settings. B-cell dynamics showed an early expansion of antigen-internalizing and antigen-presenting subsets within the first 5 days, followed by sustained active B cells throughout the 30-day simulation period (Fig 8A). Simulated antibody responses showed a peak in total IgM + IgG levels around day 15, with IgM dominating during the early phase and IgG1/IgG2 responses increasing gradually from approximately day 5 onward (Fig 8B). Helper T-cell responses peaked at approximately day 10 and remained near maximal levels, while memory Th subsets were predicted to be established early and persisted throughout the simulation period (Fig 8C). Cytokine profiling showed an IFN-γ/IL-12/IL-2-associated Th1-leaning pattern, accompanied by IL-10 and TGF-β signals, suggesting concurrent immune regulatory features within the simulated response (Fig 8D). The simulation also predicted early activation of dendritic cells and macrophages and increased antigen presentation (S7 and S8 Figs), supporting the initiation of downstream adaptive immune responses. The simulated immune-response profile provided additional computational support for retaining DRE2_SCHJA as a candidate antigen for further evaluation.

### First protein-protein docking

**Fig 9:**
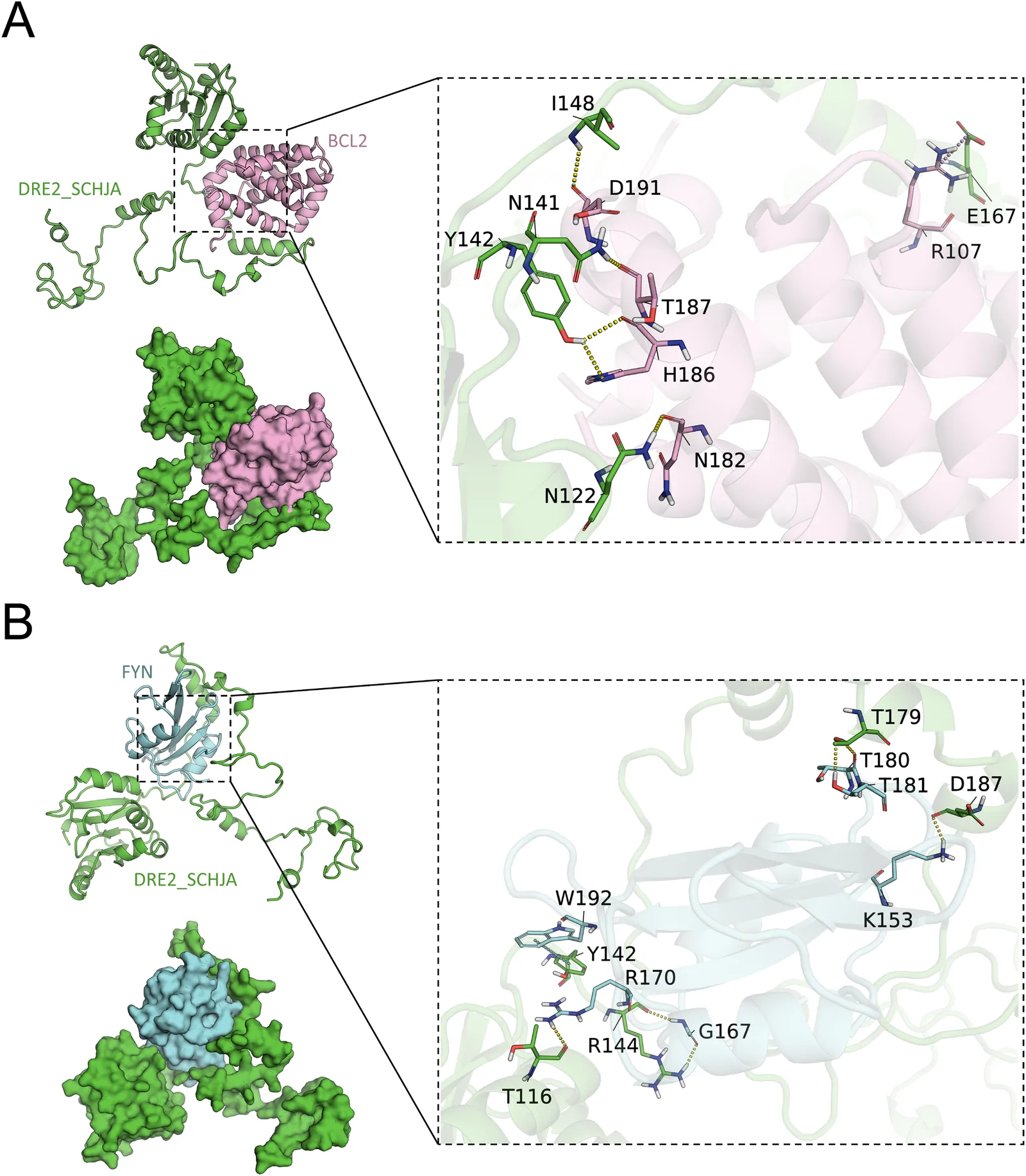
Protein–protein docking of antigen with host protein BCL2 and FYN (PDB ID: 4U1P). (A) Docking of DRE2_SCHJA–BCL2. Green protein is DRE2_SCHJA, purple protein is BCL2. Yellow dashes represent hydrogen bonds. (B) Docking of DRE2_SCHJA–FYN (PDB ID: 4U1P). Green protein is DRE2_SCHJA, blue protein is FYN. Yellow dashes represent hydrogen bonds.

**Fig 10:**
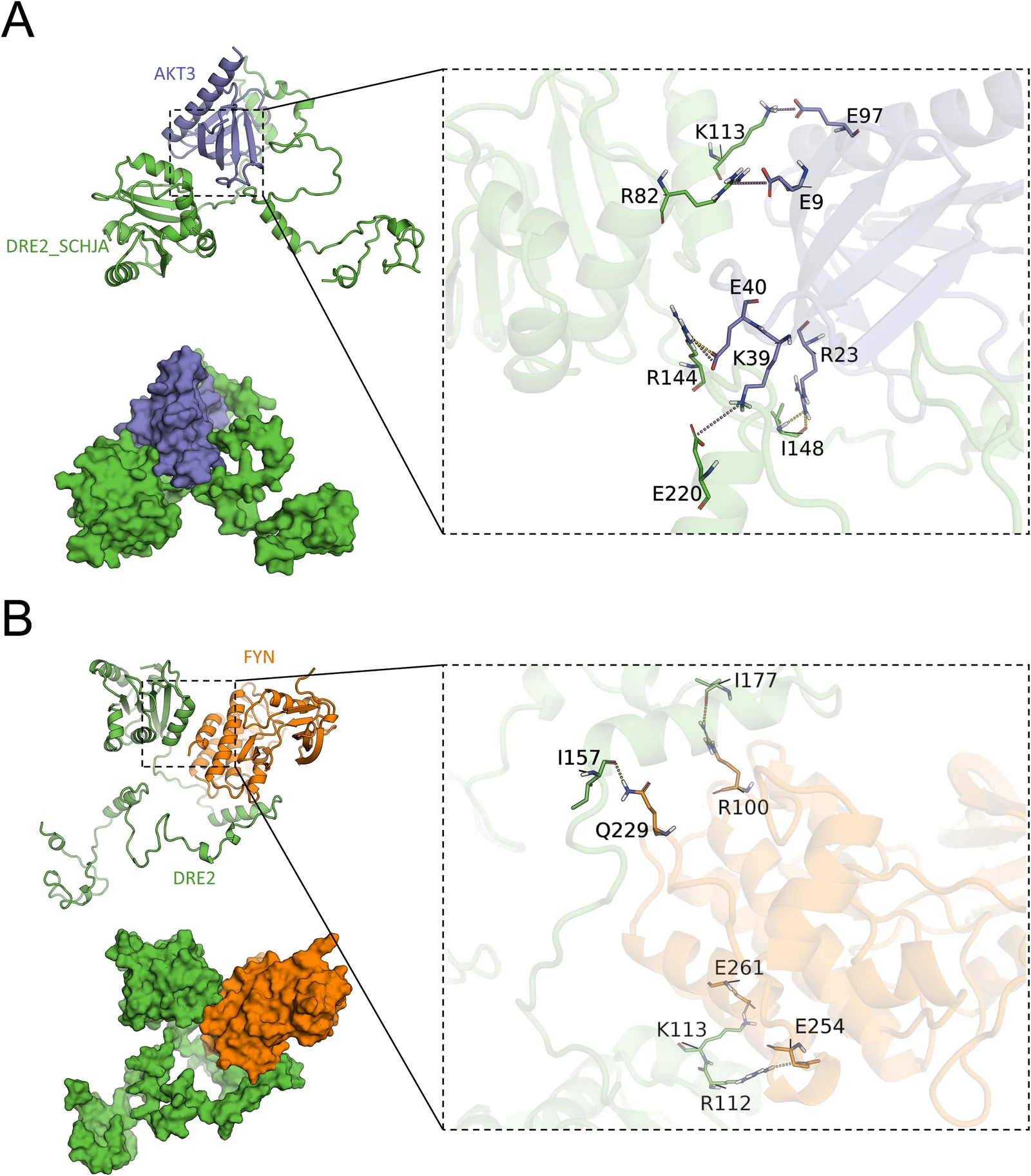
Protein–protein docking of antigen with host protein AKT3 and FYN (PDB ID: 2DQ7). (A) Docking of DRE2_SCHJA–AKT3. Green protein is DRE2_SCHJA, dark blue protein is AKT3. Yellow dashes represent hydrogen bonds, pink dashes indicate salt bridges, and blue dashes indicate π–π stacking interactions. (B) Docking of DRE2_SCHJA–FYN (PDB ID: 2DQ7). Green protein is DRE2_SCHJA, orange protein is FYN (PDB ID: 2DQ7). Yellow dashes represent hydrogen bonds.

Protein-protein docking was performed using ClusPro to explore predicted structural compatibility between DRE2_SCHJA and three host hub proteins: BCL2 (PDB ID: 8HTS), FYN (PDB ID: 4U1P), and AKT3 (PDB ID: 2X18). The DRE2_SCHJA–BCL2 complex showed a PRODIGY-predicted binding free energy (ΔG) of −12.2 kcal/mol and a docking score of −264.258, supported by five hydrogen bonds and one salt bridge (Fig 9A). The DRE2_SCHJA–AKT3 complex had a ΔG of −13.8 kcal/mol and a docking score of −199.396, with three hydrogen bonds and four salt bridges at the interface (Fig 10A). By comparison, the DRE2_SCHJA–FYN complex achieved the most favorable interaction, with a ΔG of −14.9 kcal/mol and a docking score of −252.399, and was stabilized by six hydrogen bonds and one π–π interaction (Fig 9B). Among the tested antigen–host protein pairs, DRE2_SCHJA–FYN showed the lowest predicted binding free energy, a favorable ClusPro score, and a balanced predicted interface. The DRE2_SCHJA–FYN complex was therefore prioritized for subsequent analyses.

### Molecular docking

**Fig 11:**
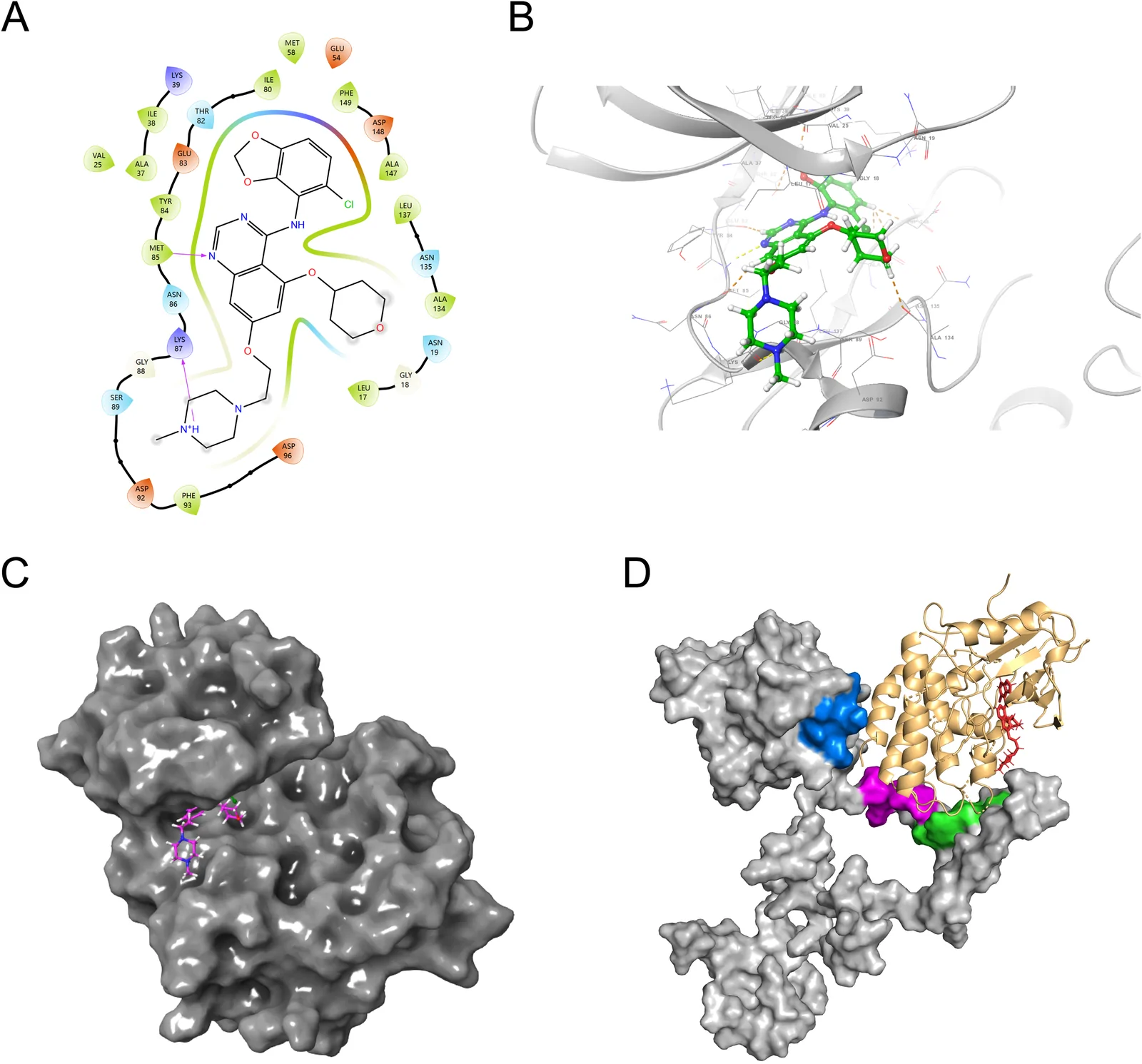
Molecular docking–based structural analysis of FYN interactions with the reference inhibitor Saracatinib and the predicted antigen DRE2_SCHJA. (A) 2D interaction map showing hydrogen bonds, hydrophobic and electrostatic contacts of Saracatinib and FYN (PDB ID: 2DQ7). (B) 3D docked conformation of Saracatinib (green sticks) in the ATP-binding pocket of FYN (PDB ID: 2DQ7). (C) Surface view of FYN (PDB ID: 2DQ7) with Saracatinib (magenta sticks) docked in the ATP-binding pocket. (D) Structural comparison of DRE2–FYN (PDB ID: 2DQ7) and Saracatinib–FYN (PDB ID: 2DQ7) complexes. FYN is shown in brown, DRE2_SCHJA in grey, and Saracatinib in red. DRE2_SCHJA binding regions are highlighted in blue, purple, and green.

Molecular docking of Saracatinib was performed using Glide in Maestro to establish a reference binding mode of FYN (PDB ID: 2DQ7). The predicted docking score (−9.251 kcal/mol) closely matched the reported value [35]. In the resulting model, the ligand reproduced key interactions reported previously [35], including hydrogen bonds with Lys87 and Asp96, hydrophobic contacts with Ile80, Tyr84, and Met85, and electrostatic stabilization by Glu83 (Fig 11A). Consistent with prior findings [35], Saracatinib occupied the ATP-binding pocket of FYN in the 3D model, stabilized through hydrogen-bonding and hydrophobic interactions (Fig 11B). The surface representation further illustrated its spatial orientation of the ligand within the binding cavity (Fig 11C). Docking of Saracatinib to FYN produced a binding pose consistent with the known inhibitor–protein interaction pattern of the FYN structure (PDB ID: 2DQ7). This result supported the use of this structure as a reference for subsequent antigen–FYN docking analyses.

### Secondary protein-protein docking

Because the FYN structure used in the initial protein–protein docking (PDB ID: 4U1P) differs from the inhibitor-bound conformation targeted by Saracatinib (PDB ID: 2DQ7), a secondary docking analysis was performed between DRE2 and the inhibitor-validated FYN structure (PDB ID: 2DQ7). This docking yielded a ClusPro docking score of −122.483 kcal/mol and a PRODIGY-predicted binding free energy of −9.3 kcal/mol. The interface was stabilized by four hydrogen bonds involving FYN residues Arg100, Arg112, Lys113, and Ile157 and DRE2 residues Ile177, Glu254, Glu261, and Gln229 (Fig 10B). Consistent with the initial protein-protein docking results, stable interfacial interactions were also observed when DRE2_SCHJA was docked to the inhibitor-validated FYN conformation (PDB ID: 2DQ7).

### Visualization

Structural alignment indicated that DRE2_SCHJA interacts with residues located in the SH2 domain of FYN (PDB ID: 2DQ7) (Fig 10B), consistent with the docking interface identified in FYN (PDB ID: 4U1P) (Fig 9B). In contrast, Saracatinib binds within the ATP-binding pocket of FYN (PDB ID: 2DQ7) (Fig 11A). The superimposed structural model further showed that the docking sites of DRE2_SCHJA and Saracatinib occupy distinct, non-overlapping regions of the FYN protein (Fig 11D).

### Motif prediction

Motif analysis predicted the presence of tyrosine-containing linear motifs in the DRE2_SCHJA sequence that are compatible with SH2 domain recognition (S1 File). When docking was performed using the FYN (PDB ID: 4U1P), several interacting residues, including R144, D187, and Y142, were located within the SH2 domain–associated region, with T116 positioned in close spatial proximity to the predicted interface. In contrast, docking using the inhibitor-validated FYN structure (PDB ID: 2DQ7) showed an interaction interface that did not spatially overlap with the predicted motif-associated region. These findings suggest that the predicted DRE2_SCHJA–FYN interface may be compatible with more than one possible SH2-associated binding mode, although this requires experimental validation.

## Discussion

This study used host single-cell transcriptomic signals from fibrotic liver tissue to guide computational antigen prioritization for SSLF. Because SSLF is shaped by host responses to parasite-derived signals, host network analysis was used to add pathology-related context to conventional antigen screening. Reverse vaccinology and docking-based assessment were then applied to prioritize parasite antigens with potential relevance to SSLF-associated host pathways. Using this framework, FYN was prioritized as a central fibrosis-associated hub within T-cell populations, and DRE2_SCHJA was prioritized as the leading parasite antigen candidate after immunogenicity- and safety-based screening. The predicted DRE2_SCHJA–FYN interaction further supported retaining this antigen for experimental validation in the immune–fibrotic context of SSLF.

To assess the disease relevance of the SSLF-prioritized gene set, comparative functional enrichment analyses were performed. Whereas HBV-only fibrosis was characterized by dominant enrichment of extracellular matrix remodeling pathways [37], the SSLF-prioritized genes were enriched for T cell–associated and intracellular signaling processes [13]. This difference suggests that the SSLF-prioritized genes may have captured immune-centered signals that were not dominant in HBV-related liver fibrosis alone. This interpretation is consistent with the inflammatory nature of SSLF, in which egg-driven immune responses interact with stromal and fibrogenic pathways. Although transcriptomic samples from patients with *S. japonicum* infection alone were unavailable, this reflects the practical difficulty of obtaining suitable human liver single-cell datasets for SSLF. In this context, the inclusion of HBV-only fibrosis and healthy controls provided an important comparative framework. Using these reference groups, the analysis helped identify signals that were not readily explained by HBV-related fibrosis alone and may represent schistosomiasis-associated signals for hypothesis generation.

Within this immune-centered network context, FYN emerged as a key upstream hub gene associated with T cell–related signaling. As a Src-family tyrosine kinase, FYN acts at an early stage of T-cell receptor signaling, linking antigen recognition–driven immune signaling to downstream processes associated with HSC activation and ECM remodeling [40]. In contrast, BCL2 and AKT3 were positioned as downstream effectors, more closely related to cell survival and metabolic adaptation than to upstream immune regulation [41]. Evidence from liver fibrosis models using Saracatinib, a Src-family kinase inhibitor, further supports the involvement of this signaling axis in fibrotic remodeling [42]. These findings support the use of FYN as a host signaling node for interpreting predicted antigen–host associations within fibrosis-associated immune networks.

Among the parasite antigens analyzed, DRE2_SCHJA was prioritized as the lead candidate because it was linked to an essential parasite pathway and met conventional immunoinformatics criteria. DRE2_SCHJA encodes an Anamorsin homolog involved in cytosolic iron–sulfur (Fe–S) cluster assembly, a pathway required for Fe–S protein maturation [43]. In schistosome infection models, Fe–S-dependent proteins such as SjSDISP have been linked to egg production, and their disruption reduced egg burden and attenuated hepatic fibrosis in vivo [44,45]. Other Fe–S–associated proteins, including SjCIAP, further support the importance of this metabolic axis in parasite development [46]. Because egg deposition is the main driver of granulomatous inflammation and liver fibrosis, disruption of Fe–S–related parasite functions may affect egg-driven pathology by impairing parasite reproduction [47]. However, this remains an indirect inference for DRE2_SCHJA. The Fe–S cluster link may support the plausibility of DRE2_SCHJA as an antigen candidate, but it does not establish functional efficacy. Experimental studies are needed to evaluate its antigen feasibility and determine whether immune targeting of this antigen affects parasite fitness, egg production, or egg-driven hepatic pathology in vivo.

Although DRE2_SCHJA is predicted to be intracellular, intracellular localization alone does not exclude immunological relevance in schistosomiasis. In chronic schistosome infection, intracellular parasite proteins may become available for host immune recognition through parasite turnover or release of parasite material in tissues. These proteins could then be taken up and processed by host antigen-presenting cells [48]. This interpretation is consistent with previous schistosomiasis vaccine studies showing that candidate antigens are not limited to classical surface-exposed proteins. Antigen candidates such as Sm14 and Sm28GST have shown immunogenicity in vaccine-development studies, supporting the principle that non-surface parasite proteins should not be excluded solely on the basis of predicted localization [49]. Nevertheless, this comparison should be interpreted cautiously, because these antigens have experimental or clinical evidence that is not yet available for DRE2_SCHJA.

The immune simulation further supported the predicted immunogenic potential of DRE2_SCHJA. The simulated profile suggested coordinated immune engagement, including antigen presentation, innate-immune activation, B-cell maturation, a Th1-leaning cytokine tendency, and regulatory cytokine signals. In SSLF, this predicted profile should be interpreted against the background of persistent egg-driven inflammation, type 2/regulatory immune polarization, macrophage–stromal crosstalk, hepatic stellate-cell activation, and extracellular-matrix deposition [50]. The predicted IFN-γ/IL-12/IL-2 pattern is compatible with a CD4 ⁺ Th1-oriented helper response, which may be relevant to SSLF because egg-driven liver fibrosis is shaped by CD4 ⁺ T-cell polarization, immune regulation, and downstream fibrogenic remodeling [51]. Sm14-based vaccine studies provide a useful mechanistic comparator, as they illustrate how antigen-specific T-cell epitopes and IFN-γ/TNF-α-associated responses may contribute to vaccine-relevant immunity in schistosomiasis [52]. In the SSLF context, this IFN-γ-related pattern is consistent with prior evidence that IFN-γ can oppose type 2 polarization and fibrogenic remodeling. It has been reported to inhibit hepatic stellate-cell activation and collagen synthesis through STAT1 signaling, and may also shape macrophage activation in ways that reduce profibrotic signaling [53]. At the same time, the presence of IL-10 and TGF-β may indicate that the simulated profile was not a purely inflammatory Th1 response. IL-10 may restrain excessive egg-driven inflammation [54], whereas persistent IL-10 and TGF-β signaling can also accompany chronic infection and fibrotic remodeling [55]. Overall, the simulated response may suggest a mixed immune profile characterized by Th1-leaning, regulatory, and B-cell-related features that are relevant to immune pathways implicated in egg-driven liver fibrosis. These findings may therefore offer a hypothesis for further experimental evaluation of DRE2_SCHJA.

Docking analysis was used to explore predicted compatibility between prioritized parasite antigens and selected host hub proteins. Among the tested host proteins, FYN showed the strongest predicted docking interaction with DRE2_SCHJA. The docking models predicted that DRE2_SCHJA may associate with SH2-related regions of FYN. In the pY-proximal mode (PDB ID: 4U1P), DRE2_SCHJA was predicted to contact Y142 and R144, which are located within the canonical phosphotyrosine recognition pocket. This prediction placed DRE2_SCHJA near the adaptor-binding region of the SH2 domain and raised a structural hypothesis related to adaptor recruitment [56]. If validated, such engagement could alter early T-cell receptor–associated signaling events [14]. In the pY-distal mode (PDB ID: 2DQ7), DRE2_SCHJA contacted residues such as Arg100 and Lys113 outside the canonical pocket. This pattern may be compatible with the broader regulatory role of SH2 domains, including allosteric or noncanonical interactions. Engagement at these distal sites may perturb the SH2–SH3 linker or the SH2-kinase interface. This could alter cross-domain communication within FYN, thereby influencing kinase activation states without directly competing for phosphotyrosine motifs [57]. However, these docking results should be regarded as structural hypotheses. They do not establish biological binding, kinase modulation, or downstream signaling effects.

The comparison with Saracatinib helped clarify this interpretation. Docking predicted that DRE2_SCHJA may bind in an SH2-associated region of FYN, whereas the Saracatinib–FYN model was centered on the ATP-binding pocket of the kinase domain [58]. This difference suggests that the DRE2_SCHJA–FYN docking result should not be interpreted as Saracatinib-like ATP-competitive kinase inhibition. Rather, it provides a computational rationale for future validation of a distinct DRE2_SCHJA–FYN interaction hypothesis. FYN participates in T-cell receptor signaling and downstream pathways such as PI3K–AKT, JAK–STAT, and Src–YAP [59], which are linked to T-cell activation, cytokine production, hepatic stellate-cell activation, and fibrotic remodeling [14]. Therefore, future experiments should test whether DRE2_SCHJA binds FYN and whether this interaction affects FYN-related immune or fibrosis-relevant signaling. At this stage, the docking analysis adds supporting computational evidence for retaining DRE2_SCHJA as a candidate antigen for further validation in the immune–fibrotic context of SSLF.

Overall, this integrative analysis supported the selection of DRE2_SCHJA as a computational candidate antigen for further validation. This selection was based on its annotated Fe–S cluster-related function, favorable in silico vaccine-candidate features, and predicted relevance to the FYN-associated immune–fibrotic context of SSLF. These findings should be interpreted as hypothesis-generating. Because human liver single-cell data from *S. japonicum*–only SSLF cases were not available, the schistosomiasis-related host signal was inferred from the available *S. japonicum*–positive liver fibrosis sample with HBV co-infection. Thus, the host-network analysis provides a computational host-pathology context to support parasite-antigen prioritization, rather than evidence for robust SSLF-specific fibrotic pathways. The predicted antigen–host interactions and immune-response patterns will require experimental validation. Future studies should validate the candidate signatures and prioritized antigens using complementary approaches, including proteomic profiling, single-cell RNA sequencing of *S. japonicum*–only cases where available, and animal models of schistosomiasis-associated granulomatous inflammation and liver fibrosis.

## Conclusion

This study used reverse vaccinology together with SSLF-relevant host pathology information to inform the screening and selection of schistosomiasis candidate antigens. DRE2_SCHJA was identified as an exploratory computational candidate that requires experimental validation. Future studies should evaluate its antigen expression, immunogenicity, and relevance to egg-driven pathology. More broadly, this integrated strategy may inform future antigen-discovery studies in schistosomiasis and other neglected tropical diseases in which host immune responses contribute to disease pathology. It may also offer a useful reference for evaluating parasite candidates in disease-relevant host contexts.

## Limitations

This study has several limitations. The first relates to the human single-cell dataset. Because human liver scRNA-seq data from *S. japonicum*–only SSLF cases were not available, this study relied on a small dataset that included one *S. japonicum*–positive liver fibrosis sample with HBV co-infection. Although HBV-related liver fibrosis and healthy samples were included as comparators, this dataset limits the ability to attribute the observed transcriptional signatures specifically to *S. japonicum* infection or SSLF. Residual effects of HBV-related liver injury, individual-sample variation, and shared fibrotic processes cannot be fully excluded.

The inclusion of only one schistosomiasis-positive individual limits the generalizability of the single-cell analysis and prevents assessment of inter-individual variation. Therefore, the differential expression and downstream analyses should be interpreted as computational observations at the cell-population level within this dataset, rather than as population-level evidence for SSLF. The predefined comparisons and alignment of results across methods helped support the internal consistency of the analysis, but validation in independent schistosomiasis cases is still needed.

The cellular scope of the analysis was also limited. This study focused mainly on T cells because of their relevance to immune-mediated fibrogenesis. However, SSLF involves interactions among multiple immune and stromal cell populations, including macrophages, hepatic stellate cells, and endothelial cells. Because these populations were not examined in detail, this study could not fully define the broader cellular networks linking egg-driven inflammation to matrix remodeling.

This study was based on computational prediction. The C-ImmSim results should be viewed as supportive in silico evidence, not as direct evidence of immunogenicity or protection. Protein–protein docking was used to explore possible binding compatibility, but molecular dynamics simulations and biochemical validation were not performed. Thus, these results should be regarded as hypothesis-generating and useful for early candidate prioritization.

Antigen selection was restricted to curated proteins to improve annotation reliability. This strategy may have excluded less characterized or novel antigens with biological relevance. Although docking suggested a possible interaction between DRE2_SCHJA and FYN, whether this interaction occurs in biological systems or affects fibrosis-related signaling remains unknown. Further experimental validation is needed to determine the expression profile of DRE2_SCHJA across parasite developmental stages, its accessibility to host immunity, and whether it can induce immune responses with protective potential against infection or egg-driven pathology.

## Supporting information

S1 Fig Quality control of single-cell RNA-seq data. (EPS)

S2 Fig Retained cell numbers across all samples after single-cell RNA-seq quality control. (EPS)

S3 Fig UMAP visualization of the top three marker genes for clusters 9–15. (TIF)

S4 Fig Comparative GO and KEGG enrichment analyses of DEGs derived from CLF–HC (HBV-related liver fibrosis) and SJ–HC (*Schistosoma japonicum*–HBV co-infection) contrasts. (EPS)

S5 Fig STRING-based protein–protein interaction network. (EPS)

S6 Fig Overlap of UniProt-reviewed *Schistosoma japonicum* protein entries annotated in the three Gene Ontology domains: biological process, cellular component, and molecular function. (EPS)

S7 Fig C-ImmSim-predicted dendritic-cell population after simulated vaccination. (EPS)

S8 Fig C-ImmSim-predicted macrophage population after simulated vaccination. (EPS)

S1 Table Stepwise analytical pipeline for hub gene prioritization in SSLF. (DOCX)

S2 Table Fifty-two UniProt-reviewed *Schistosoma japonicum* protein entries used for antigen screening. (DOCX)

S3 Table UniProt-reviewed *Schistosoma japonicum* protein entries annotated in at least two Gene Ontology domains. (DOCX)

S1 File Motif prediction of DRE2_SCHJA. (DOCX)
