# Supplementary material for: Host immune network-guided reverse vaccinology approach for prioritizing candidate antigens in Schistosoma japonicum–associated liver fibrosis
Source: PLoS Negl Trop Dis. 2026 Aug 17;20(8):e0013519. doi: 10.1371/journal.pntd.0013519 (PMC13492981; doi:10.1371/journal.pntd.0013519)
Supplement: Supplementary file 1 [file pntd.0013519.s009.docx]

Table S1. Stepwise analytical pipeline for hub gene prioritization in *Schistosoma japonicum*–associated liver fibrosis

| Step | Analysis | Sub analysis | Objective | Cut-off / Method clarification | **Input** | **Output** |
| --- | --- | --- | --- | --- | --- | --- |
| 1 | Single cell RNA-seq | - Quality control - Dimensionality reduction, - clustering, and annotation | To establish fibrotic and baseline cellular reference profiles for comparative analysis under SJ–HBV co-infection. | - Cells retained with 200–10,000 detected genes and <25% mitochondrial content - Doublets removed using DoubletFinder - Standard Seurat normalization, HVG selection, PCA, clustering, and UMAP/t-SNE were applied - Cell-type annotation using SingleR and manual marker-based refinement | - Cells before QC:   CLF1:3857  CLF2:3114  CLF3:4895  HC1:4285  HC2:1443  SJ1:7914 | - Cells after QC:   CLF1:3063  CLF2:2966  CLF3:4507  HC1:3429  HC2:1333  SJ1:7374 |
| 2 | hdWGCNA | Gene co-expression module construction | To construct gene co-expression modules from scRNA-seq data using metacell aggregation. | - Genes expressed in ≥5% of cells - Metacell construction (k = 25, max_shared = 10) - Signed hybrid network with soft-threshold power = 3 (scale-free R² ≈ 0.95). | Gene-by-metacell expression matrix | Gene co-expression modules and module eigengenes (nine modules, including the grey module; 3,432 genes) |
|  |  | Module–condition association analysis | To identify WGCNA modules preferentially associated with the SJ condition compared with CLF and HC. | Module–condition Pearson correlation analysis (SJ, CLF, HC), p < 0.05 | Module eigengenes of eight non-grey WGCNA modules | Module–condition correlation profiles for eight non-grey WGCNA modules, with identification of the module most strongly associated with the SJ condition (turquoise=1493 genes) |
|  |  | Module–cell type association analysis | To identify the cell population most strongly associated with the SJ-associated WGCNA module. | Module–cell type Pearson correlation, p < 0.05; module activity assessed by UCell. | Module eigengenes of eight non-grey WGCNA modules | Module–cell type correlation profiles across thirteen cell populations, with identification of the T-cell population most strongly associated with the SJ-associated WGCNA module. |
| 3 | Differentially expressed genes (DEGs) | Multi-group T-cell DEG contrasts (SJ–HC; CLF–HC; SJ –CLF) | To identify differentially expressed genes across SJ, CLF, and HC groups within the T-cell population. | Adjusted p < 0.05, \|log₂FC\| ≥ 1 | Normalized T-cell gene expression matrix | T-cell DEG lists for SJ–HC(2489 genes), CLF–HC(1030 genes), and SJ–CLF(1187 genes) |
|  |  | Exclusion of CLF–HC shared DEGs from SJ–HC DEGs | To remove genes commonly differentially expressed in both SJ–HC and CLF–HC comparisons. | Venn-based exclusion of CLF–HC–shared DEGs from SJ–HC DEGs. | - SJ–HC DEG list (2489 genes) - CLF–HC DEG list(1030 genes) | SJ-biased DEG set (1769 genes) |
|  |  | Intersection of filtered SJ–HC DEGs with SJ–CLF DEGs and SJ-associated WGCNA module | To define an SSLF-prioritized candidate gene set by intersecting the SJ-biased DEG set with SJ vs CLF DEGs and genes from the SJ-associated WGCNA module | Venn-based intersetion | - SJ-biased DEG set (1769 genes) - SJ–CLF DEG list (1187 genes) - Genes assigned to the SJ-associated WGCNA module (1493 genes) | SSLF-prioritized candidate gene set (219 genes) |
| 4 | Comparative GO/KEGG enrichment analysis | - | To assess whether SSLF-prioritized genes display functional patterns distinct from HBV-only fibrosis | Identical enrichment pipeline and thresholds applied across contrasts: Adjusted p < 0.05, \|log₂FC\| ≥ 1 | T-cell DEGs from CLF–HC(1030 genes), SJ–HC(2489 genes), and SSLF-prioritized gene set (219 genes) | Functional profiles showing reduced representation of HBV-associated fibrotic signatures and enrichment of immune-regulatory pathways |
| 5 | PPI and hub genes | STRING PPI construction | To place SSLF-enriched DEGs in a curated PPI context for network-based prioritization. | Interaction score: 0.4 | SSLF-prioritized candidate gene set (219 genes) | 152 interconnected proteins |
|  |  | CytoHubba centrality ranking | To prioritize genes with high network centrality across twelve metrics, capturing broadly connected nodes in the SSLF-associated PPI network. | Twelve CytoHubba algorithms: MCC, DMNC, MNC, Degree, EPC, BottleNeck, EcCentricity, Closeness, Radiality, Betweenness, Stress, and Clustering Coefficient | 152 interconnected proteins | Six highly central proteins(AKT3, ANK3, ATM, BCL2, CUL3, and FYN) |
|  |  | MCODE module detection | To identify densely connected subnetworks within the SSLF-associated PPI network and prioritize the top-three ranking modules based on MCODE scores. | degree cutoff = 2, node score cutoff = 0.2, k-core = 2, maximum depth = 100; haircut enabled, fluff disabled | 152 interconnected proteins | Top-three ranking modules |
|  |  | Hub gene definition | To integrate global node centrality with local network modularity, cytoHubba-prioritized central PPI nodes were intersected with genes from the combined high-scoring MCODE clusters to define SSLF hub candidates. | - | - Six highly central proteins - Gene set derived from the combined top-three scoring MCODE modules | SSLF-specific hub genes (AKT3, BCL2, FYN) |
