# Supplementary material for: Host immune network-guided reverse vaccinology approach for prioritizing candidate antigens in Schistosoma japonicum–associated liver fibrosis
Source: PLoS Negl Trop Dis. 2026 Aug 17;20(8):e0013519. doi: 10.1371/journal.pntd.0013519 (PMC13492981; doi:10.1371/journal.pntd.0013519)
Supplement: Supplementary file 2 [file pntd.0013519.s010.docx]

S2 Table. Fifty-two *S.japonicum* antigen entries

| Ontology | Items | Antigen numbers | Antigen list |
| --- | --- | --- | --- |
| Biological process(BP) | Biological regulation | 6 | PPIE_SCHJA, PPIB_SCHJA, ATAT_SCHJA, EIF3M_SCHJA,HGLB_SCHJA, CH10_SCHJA |
|  | Cellular process | 21 | GST28_SCHJA, GST26_SCHJA, PPIE_SCHJA, CYSP_SCHJA, MYSP_SCHJA, HGLB_SCHJA, DRE2_SCHJA, PURA_SCHJA, ENO_SCHJA, KYNU_SCHJA, TPIS_SCHJA, PPIB_SCHJA, RL24_SCHJA, CDO_SCHJA, CIAO1_SCHJA, RS3A_SCHJA, RSSA_SCHJA, CH10_SCHJA, ATAT_SCHJA, EIF3M_SCHJA, HSP70_SCHJA |
|  | Developmental process | 3 | MYSP_SCHJA, ATAT_SCHJA, EIF3M_SCHJA |
|  | Regulation of biological process | 5 | PPIE_SCHJA, PPIB_SCHJA, ATAT_SCHJA, EIF3M_SCHJA, HGLB_SCHJA |
|  | Multicellular organismal process | 2 | ATAT_SCHJA, HGLB_SCHJA |
| Cellular component (CC) | Extracellular region | 13 | ANX13_SCHJA, SJ144_SCHJA, SJ047_SCHJA, SJ380_SCHJA, SJ965_SCHJA, SJ643_SCHJA, SJ381_SCHJA, CP422_SCHJA, SJ800_SCHJA, SJ803_SCHJA, SJ711_SCHJA, SJ766_SCHJA, SJ055_SCHJA |
|  | Extracellular space | 1 | ANX13_SCHJA |
|  | Cytoplasm | 23 | ANX13_SCHJA, FABP_SCHJA, PPIE_SCHJA, MYSP_SCHJA, HGLB_SCHJA, DRE2_SCHJA, PURA_SCHJA, ENO_SCHJA, KYNU_SCHJA, TPIS_SCHJA, PPIB_SCHJA, RL24_SCHJA, TCTP_SCHJA, SORCN_SCHJA, TOR_SCHJA, CIAO1_SCHJA, RS3A_SCHJA, IFT46_SCHJA, RSSA_SCHJA, CH10_SCHJA, EIF3M_SCHJA |
|  | Actin filament bundle | 1 | ACTA_MOUSE |
|  | Actomyosin | 1 | ACTA_MOUSE |
|  | Membrane | 4 | TOR_SCHJA, IM25_SCHJA, IM23_SCHJA, ANX13_SCHJA |
|  | Cell periphery | 5 | ANX13_SCHJA, IM23_SCHJA, TOR_SCHJA |
|  | Supramolecular complex | 3 | MYSP_SCHJA, ATAT_SCHJA |
|  | Endomembrane system | 2 | PPIB_SCHJA |
|  | External encapsulating structure | 1 | ACTA_MOUSE |
|  | Catalytic complex | 1 | ENO_SCHJA |
|  | Myosin complex | 1 | MYSP_SCHJA |
|  | Intracellular protein-containing complex | 1 | CIAO1_SCHJA |
|  | Ribonucleoprotein complex | 4 | RL24_SCHJA, RS3A_SCHJA, RSSA_SCHJA, EIF3M_SCHJA |
| Molecular function(MF) | binding | 17 | ANX13_SCHJA, FABP_SCHJA, PPIE_SCHJA, DRE2_SCHJA, PURA_SCHJA, ENO_SCHJA, KYNU_SCHJA, PPIB_SCHJA, ACTA_MOUSE, SEPT4_MOUSE, TCTP_SCHJA, RL24_SCHJA, SORCN_SCHJA, CDO_SCHJA, CH10_SCHJA, EIF3M_SCHJA, HSP70_SCHJA |
|  | catalytic activity | 13 | GST28_SCHJA, GST26_SCHJA, PPIE_SCHJA, PURA_SCHJA, CYSP_SCHJA, HGLB_SCHJA, ENO_SCHJA, KYNU_SCHJA, TPIS_SCHJA, PPIB_SCHJA, ACTA_MOUSE, CDO_SCHJA, ATAT_SCHJA |
|  | protein folding chaperone | 2 | CH10_SCHJA, HSP70_SCHJA |
|  | structural molecule activity | 3 | RL24_SCHJA, RS3A_SCHJA, RSSA_SCHJA |
