# Supplementary material for: Host immune network-guided reverse vaccinology approach for prioritizing candidate antigens in Schistosoma japonicum–associated liver fibrosis
Source: PLoS Negl Trop Dis. 2026 Aug 17;20(8):e0013519. doi: 10.1371/journal.pntd.0013519 (PMC13492981; doi:10.1371/journal.pntd.0013519)
Supplement: Supplementary file 3 [file pntd.0013519.s011.docx]

Table S3. The overlapping antigens among BP, CC and MF

| Intersection | Antigen numbers | Antigen list |
| --- | --- | --- |
| BP ∩ CC ∩ MF | 14 | PPIE_SCHJA, PPIB_SCHJA, HGLB_SCHJA, DRE2_SCHJA, PURA_SCHJA, ENO_SCHJA, KYNU_SCHJA, TPIS_SCHJA, RL24_SCHJA, RS3A_SCHJA, RSSA_SCHJA, CH10_SCHJA, ATAT_SCHJA, EIF3M_SCHJA |
| BP ∩ MF | 5 | GST28_SCHJA, GST26_SCHJA, CYSP_SCHJA, CDO_SCHJA, HSP70_SCHJA |
| CC ∩ MF | 4 | ANX13_SCHJA, FABP_SCHJA, TCTP_SCHJA, SORCN_SCHJA |
| BP ∩ CC | 2 | MYSP_SCHJA, CIAO1_SCHJA |
