# Supplementary material for: Host immune network-guided reverse vaccinology approach for prioritizing candidate antigens in Schistosoma japonicum–associated liver fibrosis
Source: PLoS Negl Trop Dis. 2026 Aug 17;20(8):e0013519. doi: 10.1371/journal.pntd.0013519 (PMC13492981; doi:10.1371/journal.pntd.0013519)
Supplement: Supplementary file 4 [file pntd.0013519.s012.docx]

Supplementary File S1. Predicted Eukaryotic Linear Motifs (ELMs) Identified in DRE2_SCHJA

| Elm Name | Instances (Matched Sequence) | Positions | View in Jmol | Elm Description | Cell Compartment | Pattern | PHI-Blast Instance Mapping | Structural Filter Info | Probability |
| --- | --- | --- | --- | --- | --- | --- | --- | --- | --- |
| CLV_C14_Caspase3-7 | ENVDN | 162-166 [A] | - | Caspase-3 and Caspase-7 cleavage site. | cytosol, nucleus | [DSTE][^P][^DEWHFYC]D[GSAN] | - | - | 3.094e-03 |
| CLV_NRD_NRD_1 | FRK; KRK | 111-113 [A]; 206-208 [A] | - | N-Arg dibasic convertase (NRD/Nardilysin) cleavage site (X- | -R-K or R- | -R-X). | extracellular, Golgi apparatus, cell surface | (.RK)\|(RR[^KR]) | - |
| DEG_APCC_DBOX_1 | ERGLLSNLS | 81-89 [A] | - | RxxL-based motif targeting protein for APC/C-mediated degradation. | nucleus, cytosol | .R..L..[LIVM]. | - | - | 7.677e-04 |
| DEG_Nend_UBRbox_2 | MEP | 1-3 [A] | - | N-terminal degron binding UBR-box of N-recognins. | cytosol | ^M{0,1}([ED]). | - | - | 2.537e-04 |
| DOC_CKS1_1 | YVTPLA | 188-193 [A] | - | Phospho-dependent docking motif for CDK substrates via Cks1. | cytosol, nucleus | [MPVLIFWYQ].(T)P.. | - | - | 1.991e-03 |
| DOC_CYCLIN_RxL_1 | THVKKLHFENLE; TLERGLLSNLS; SFRKNLTLS | 37-48 [A]; 79-89 [A]; 110-118 [A] | - | Cyclin/CDK RxL docking motifs. | nucleus, membrane, cytosol, centriole, Cajal body | (. | ([KRH].{0,3}))[^EDWNSG][^D][RK][^D]L.{0,1}[FL].{0,3}[EDST] | - | - |
| DOC_PP1_RVXF_1 | HVKKLHFE; VKKLHFE | 38-45 [A]; 39-45 [A] | - | PP1 catalytic subunit docking motif. | nucleus, PP1 complex, cytosol | ..[RK].{0,1}[VIL][^P][FW]. | - | - | 8.301e-04 |
| DOC_USP7_MATH_1 | AAVST; AKNSI | 33-37 [A]; 200-204 [A] | - | USP7 MATH domain binding motif. | nucleus | [PA][^P][^FYWIL]S[^P] | - | - | 1.239e-02 |
| DOC_USP7_UBL2_3 | KRACK | 208-212 [A] | - | USP7 CTD binding motif. | nucleus | K...K | - | - | 3.742e-03 |
| DOC_WW_Pin1_4 | DYVTPL | 187-192 [A] | - | Pin1 WW domain interaction motif. | cytosol, nucleus | ...([ST])P. | - | - | 1.543e-02 |
| LIG_14-3-3_CanoR_1 | RKNLTLS; RCSTCP | 112-118 [A]; 247-252 [A] | - | Canonical 14-3-3 phospho-binding motif. | cytosol, nucleus, plasma membrane | R[^DE]{0,2}[^DEPG](https://chatgpt.com/g/g-p-693a25a8619c81919e70e45a46095271-target-paper-revision/c/%5BST%5D) | - | - | 4.477e-03 |
| LIG_BRCT_BRCA1_1 | ISSQF | 56-60 [A] | - | BRCA1 BRCT domain phospho-motif. | nucleus, BRCA1-BARD1 complex | .(S)..F | - | - | 1.912e-03 |
| LIG_LIR_Gen_1 | EDWNSFRKNL; DWNSFRKNL; EAAWENVDNE | 106-115 [A]; 107-115 [A]; 158-167 [A] | - | Canonical LC3/Atg8-interacting region (LIR). | cytosol, late endosome membrane | [EDST].{0,2}[WFY][^RKPGWFY][^PG][ILVFM] | - | - | 3.631e-03 |
| LIG_SH2_SFK_2 | SGYVNPY; SDYVTPL | 118-124 [A]; 186-192 [A] | - | Src-family kinase SH2 binding motif. | cytosol, plasma membrane | [^KR][^KR](https://chatgpt.com/g/g-p-693a25a8619c81919e70e45a46095271-target-paper-revision/c/Y)... | - | - | 8.808e-04 |
| LIG_SH2_STAT5 | YVVD; YVNP; YTRG; YVTP; YLGD | 4-7; 120-123; 142-145; 188-191; 241-244 [A] | - | STAT5 SH2 domain binding motif. | cytosol | (Y)[VLTFIC].. | - | - | 3.296e-03 |
| TRG_NLS_MonoCore_2 | GKRKRA | 205-210 [A] | - | Classical monopartite nuclear localization signal. | nucleus | [^DE]((K[RK]) | (RK))[KRP][KR][^DE] | - | - |
| TRG_NLS_MonoExtN_4 | KRKRAC | 206-211 [A] | - | Extended monopartite NLS. | nucleus | ([PKR].{0,1}[^DE])((K[RK]) | (RK)) | - | - |
